# Synthesis of novel SRPK1-targeting hybrid compounds incorporating antimetabolites for cancer therapy

**DOI:** 10.1039/d5md00731c

**Published:** 2025-09-29

**Authors:** George Leonidis, Ioanna Sigala, Michail Spathakis, George Kolios, Thomas Giannakouros, Eleni Nikolakaki, Vasiliki Sarli

**Affiliations:** a Department of Chemistry, Aristotle University of Thessaloniki Greece sarli@chem.auth.gr; b Department of Medicine, Democritus University of Thrace Greece

## Abstract

Serine/arginine protein kinase 1 (SRPK1) plays a pivotal role in the phosphorylation of SR/RS domain-containing proteins, which are involved in various cellular processes. Its overexpression has been associated with the progression of various malignancies, positioning SRPK1 as a promising target for cancer treatment. In this study, we report the design, synthesis, and preliminary biological evaluation of two hybrid molecules, geo15 and geo140, which combine known SRPK1 inhibitors with the antimetabolites gemcitabine and 5-fluorouracil (5-FU), respectively. These conjugates were synthesized to assess whether hybridization enhances potency compared to the parent compounds, and to investigate potential novel mechanisms of action. *In vitro* assays were performed to evaluate SRPK1 inhibition and antiproliferative activity in selected cancer cell lines. Among the tested compounds, the JH-VII-139-1-based hybrid geo140 exhibited notable SRPK1 inhibitory potency and cytotoxic effects, demonstrating a favorable profile for further optimization. Interestingly, treatment with geo140 did not appear to alter the overall SRPK1 distribution in interphase cells but resulted in a notable increase of mitotic cells that displayed a substantial accumulation of SRPK1, thus suggesting that the hybrid compound may have an impact on cell cycle progression. This work supports the potential of molecular hybridization as a strategy for the development of novel SRPK1-targeting anticancer agents.

## Abstract 2

Geo140, the JH-VII-139-1-based hybrid with 5-FU exhibits notable SRPK1 inhibitory potency and cytotoxic effects against HeLa and K562 cancer cells.

## Graphical abstract

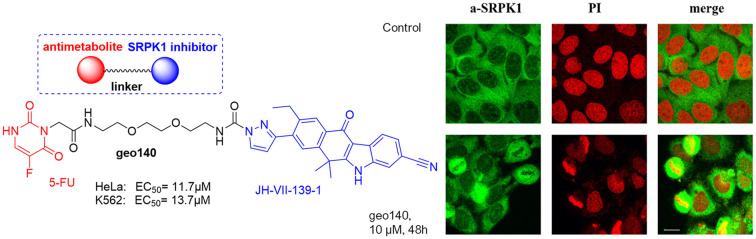

## Introduction

Serine/arginine protein kinase 1 (SRPK1) is widely known as a splice factor kinase due to its decisive involvement in the regulation of various steps of mRNA splicing *via* the phosphorylation of SR splicing factors.^1^ However, as the mammalian genome encodes for more than 100 serine-arginine rich proteins -half of which are unrelated to mRNA processing-, the kinase was also shown to regulate diverse cellular activities.^2,3^ These fundamentally important functions render SRPK1 essential for viability of proliferating cells. Many reports suggest that there is a correlation between SRPK1 overexpression and higher tumor staging, grading, metastasis and shorter survival.^4,5^ Consequently, targeting SRPK1 has emerged as a promising therapeutic strategy. Indeed, downregulation of SRPK1 in cancer cells that exhibit high SRPK1 levels suppressed their growth and affected diverse processes heterogeneously, depending on the oncogenic context.^4–6^ For example, vascular endothelial growth factor A (VEGF-A) can produce both proangiogenic and antiangiogenic isoforms; thus, a shift in the splicing machinery towards the production of the former, *via* upregulation of SRPK1, enhanced angiogenesis.^7,8^ Wang *et al.* also proposed that aberrant SRPK1 expression induced constitutive Akt activation, thus implying that SRPK1 can mediate tumorigenesis independently of its splicing effects, by modulating signaling pathways such as Akt.^9^ In addition, targeting SRPK1 resulted in enhanced sensitivity to platinum-based chemotherapy in some types of cancer.^2,4^ A wide range of SRPK1 inhibitors have been developed, including SRPIN340, SPHINX, and the more recent JH-VII series compounds, all of which demonstrate potent inhibitory activity against SRPK1.^4,10–12^ The growing number of small-molecule inhibitors underlines the pharmacological relevance of SRPK1 as a cancer treatment target.

Antimetabolites such as 5-fluorouracil (5-FU) and gemcitabine are cornerstone chemotherapeutic agents used in the treatment of a wide range of solid tumours and hematologic malignancies. These drugs interfere with nucleotide metabolism and DNA synthesis, leading to cell cycle arrest and apoptosis in rapidly dividing cells. Despite their efficacy, limitations such as systemic toxicity, resistance development, and off-target effects often reduce therapeutic outcomes. To overcome these issues, there is growing interest in conjugation strategies that combine antimetabolites with targeting moieties or other pharmacophores to enhance selectivity and synergistic activity.

**Fig. 1:**
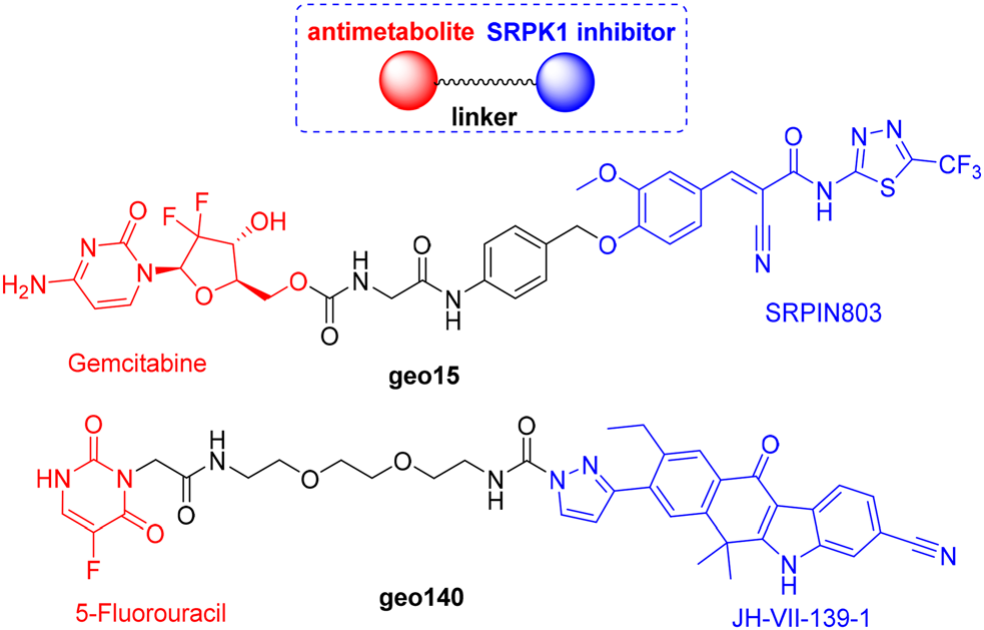
Structures of novel SRPK1-targeting hybrids that incorporate antimetabolites.

In previous studies, our group developed peptide–drug conjugates incorporating the oligopeptide c(RGDyK), an integrin α_v_β_3_ ligand, for the targeted delivery of SRPK1 inhibitors (SRPIN803, JH-VII-139-1).^13–16^ These studies yielded promising results, demonstrating both the synergistic effects of the two pharmacophores and novel applications. As a following step, we sought to explore the concept of molecular hybridization by covalently linking SRPK1 inhibitors with antimetabolites, inspired by previous research showing that targeting of SRPK1 results in enhanced sensitivity to platinum-based drugs in some types of cancer.^2,4^ Our aim was to evaluate whether these hybrids could exhibit enhanced potency, improved physicochemical properties, or new mechanisms of action compared to the parent pharmacophores. Specifically, SRPIN803 was coupled with gemcitabine, and JH-VII-139-1 with 5-fluorouracil, producing the hybrid compounds geo15 and geo140, respectively (Fig. 1). Herein, we describe the synthesis of these hybrids and evaluate their chemostability in buffer media, DMEM, and human plasma. Additionally, we report on their biological properties, including cellular viability, SRPK1 inhibition, subcellular distribution of the kinase, and a potential mode of action of geo140.

## Synthesis of SRPK1-targeting hybrids

### Synthesis of conjugate geo15

**Fig. 2:**
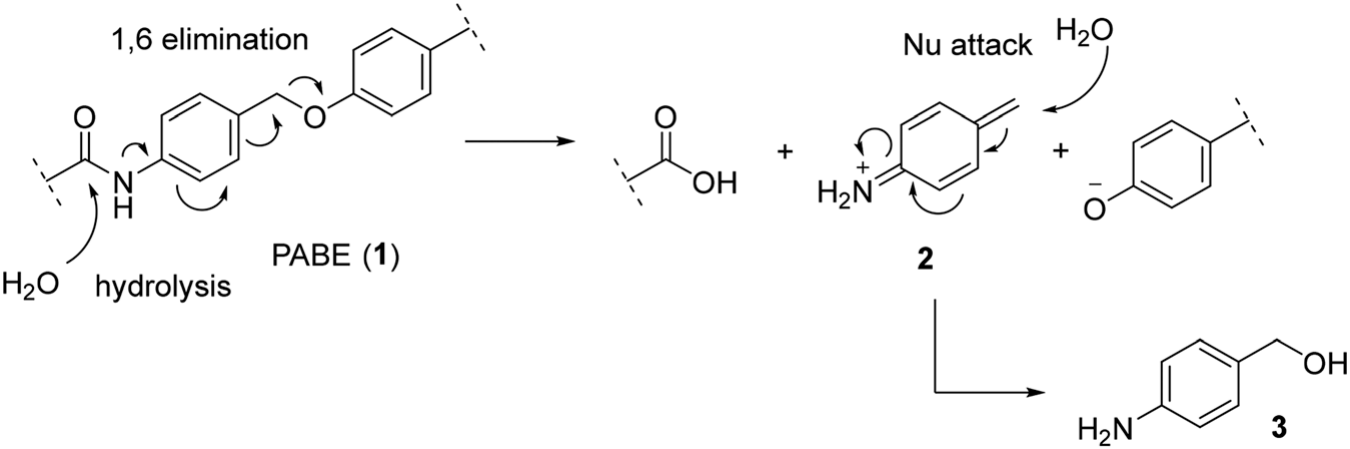
Self-immolation mechanism of PABE linkers.

Based on our prior experience synthesizing two SRPIN803-c(RGDyK) conjugates bearing non-cleavable ether and secondary carbamate linkers, our next goal was to design a linker with sufficient stability for attachment to the phenolic group of SRPIN803 without being readily hydrolyzed.^15^ We selected *para*-aminobenzyl alcohol (3), which is converted to *para*-aminobenzyl ether (PABE, 1) upon coupling with phenol and carboxylic acid derivatives. PABE is a cleavable linker capable of self-immolation *via* a 1,6-elimination mechanism following hydrolysis at the aniline end (Fig. 2).^17^ Recent literature shows that PABE linkers are used in various hybrid drugs containing phenolic ends, which are often susceptible to hydrolysis.^18^

**Scheme 1:**
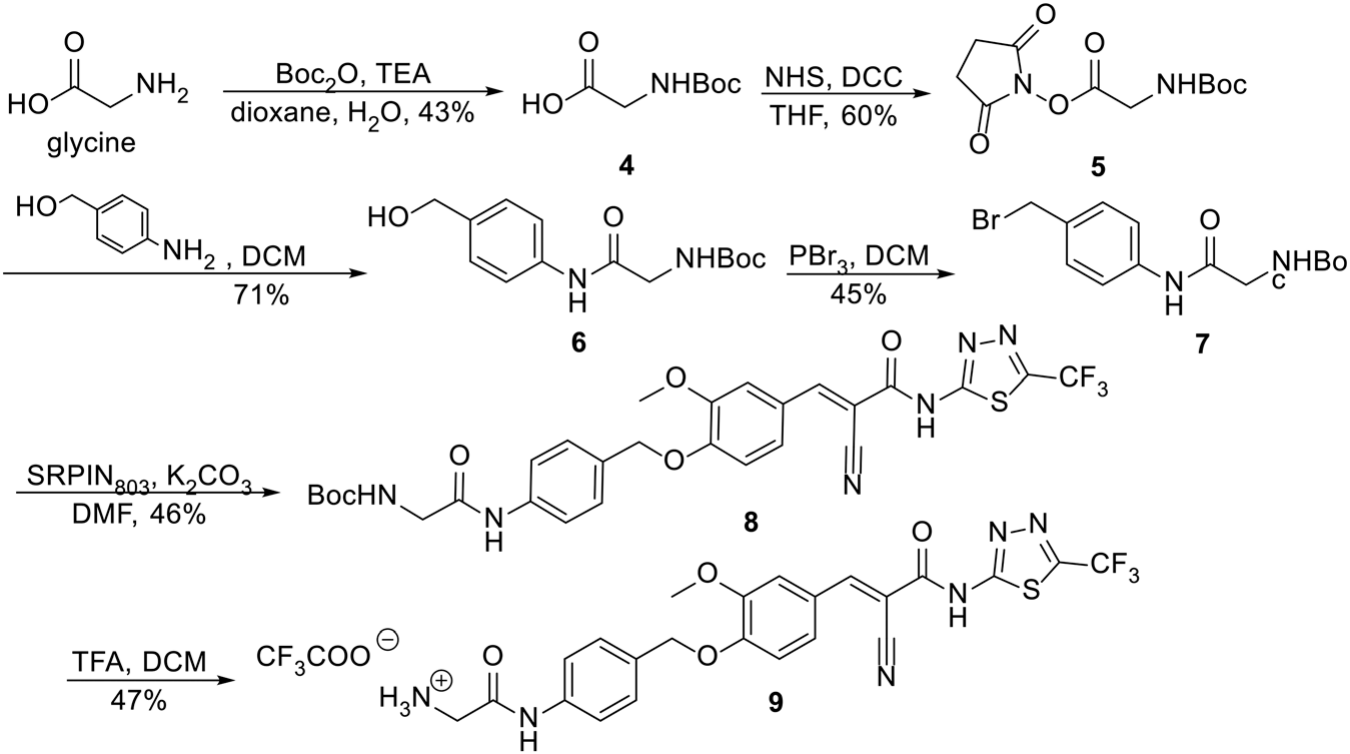
Synthetic route of SRPIN803 derivative 9.

To facilitate the coupling reactions of the PABE linker, a glycine moiety to the aniline side was attached through an amidation reaction, introducing a more reactive primary amine that does not interfere with the self-immolation mechanism. The synthetic route started with the preparation of bocylated glycine 4 from commercially available glycine, which reacted with Boc_2_O under basic conditions to yield the desired product with 43% yield (Scheme 1). Before the amidation step, compound 4 was activated with *N*-hydroxysuccinimide and DCC, producing the isolable activated ester 5 in 60% yield. Ester 5 and *para*-aminobenzyl alcohol then readily reacted to form the desired product 6 in 71% yield. Compound 6 was subsequently treated with phosphorus tribromide in diethyl ether, resulting in the unstable bromide intermediate 7 in 45% yield. Bromide 7 was then immediately reacted with SRPIN803 in a Williamson ether synthesis, yielding the intermediate 8 with a 46% yield. The synthetic route ended with Boc deprotection under acidic conditions, isolating the SRPIN803 derivative 9 as a TFA salt in 47% yield.

**Scheme 2:**
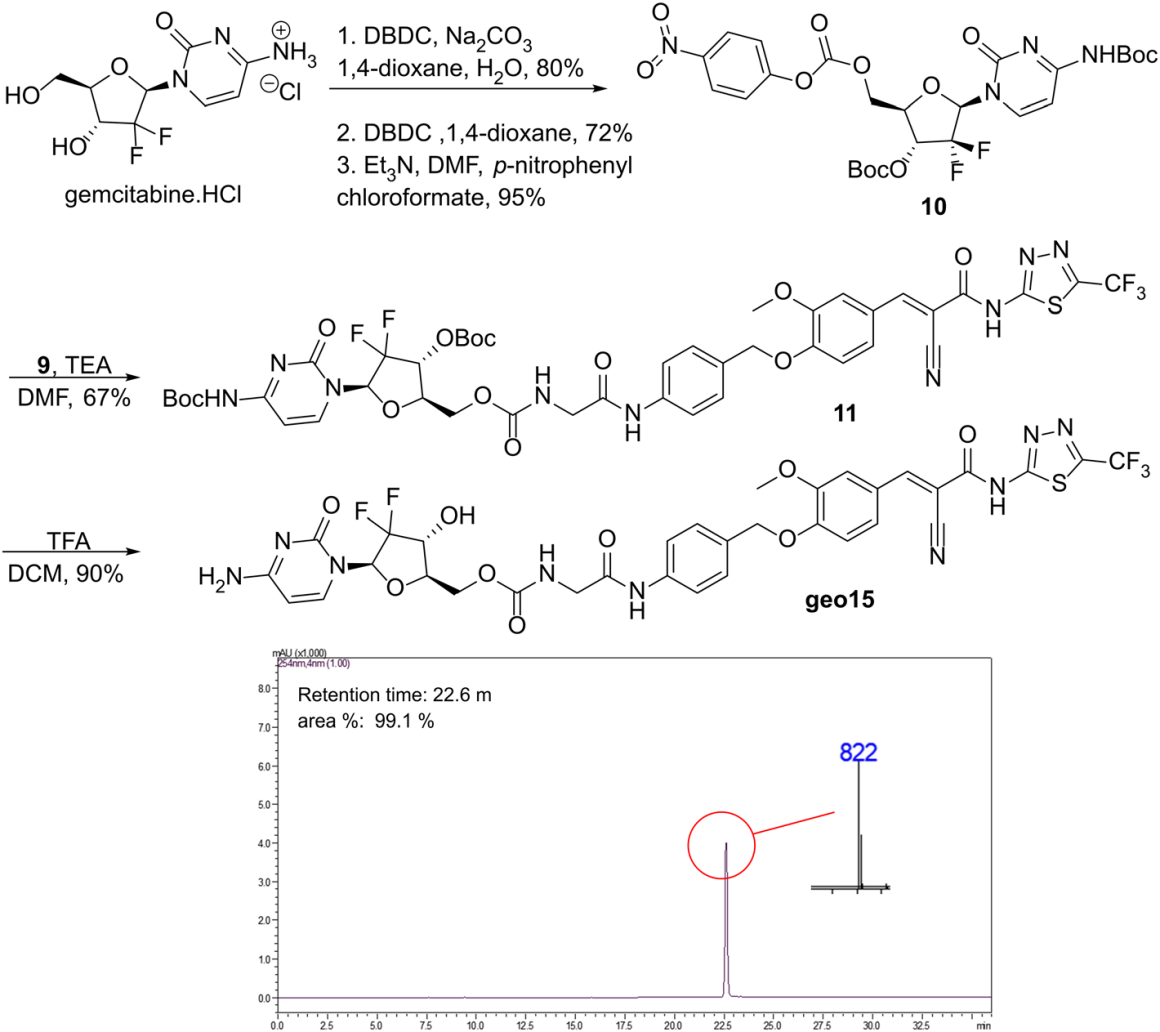
Synthesis of conjugate geo15 and its LC-MS elution chromatogram. Method: 3.

With the TFA salt 9 in hand, we proceeded with a coupling reaction with the activated gemcitabine 10, yielding the Boc-protected SRPIN803-gem conjugate 11 in 67% yield (Scheme 2). As reported in our previous work, activated gemcitabine 10 was obtained through consecutive protection and activation reactions of gemcitabine hydrochloride.^19^ The final desired conjugate geo15, was obtained after deprotection of the two Boc groups on gemcitabine using a solution of TFA in DCM. Conjugate geo15 was isolated by HPLC as a yellow, fluorescent solid with purity over 99% and yield 90%. The calculated exact mass for the [M + H]^+^ ion of the final conjugate is 822.1724, and a mass of 822.0 was observed in the mass spectrometer.

### Synthesis of conjugate geo140

**Scheme 3:**
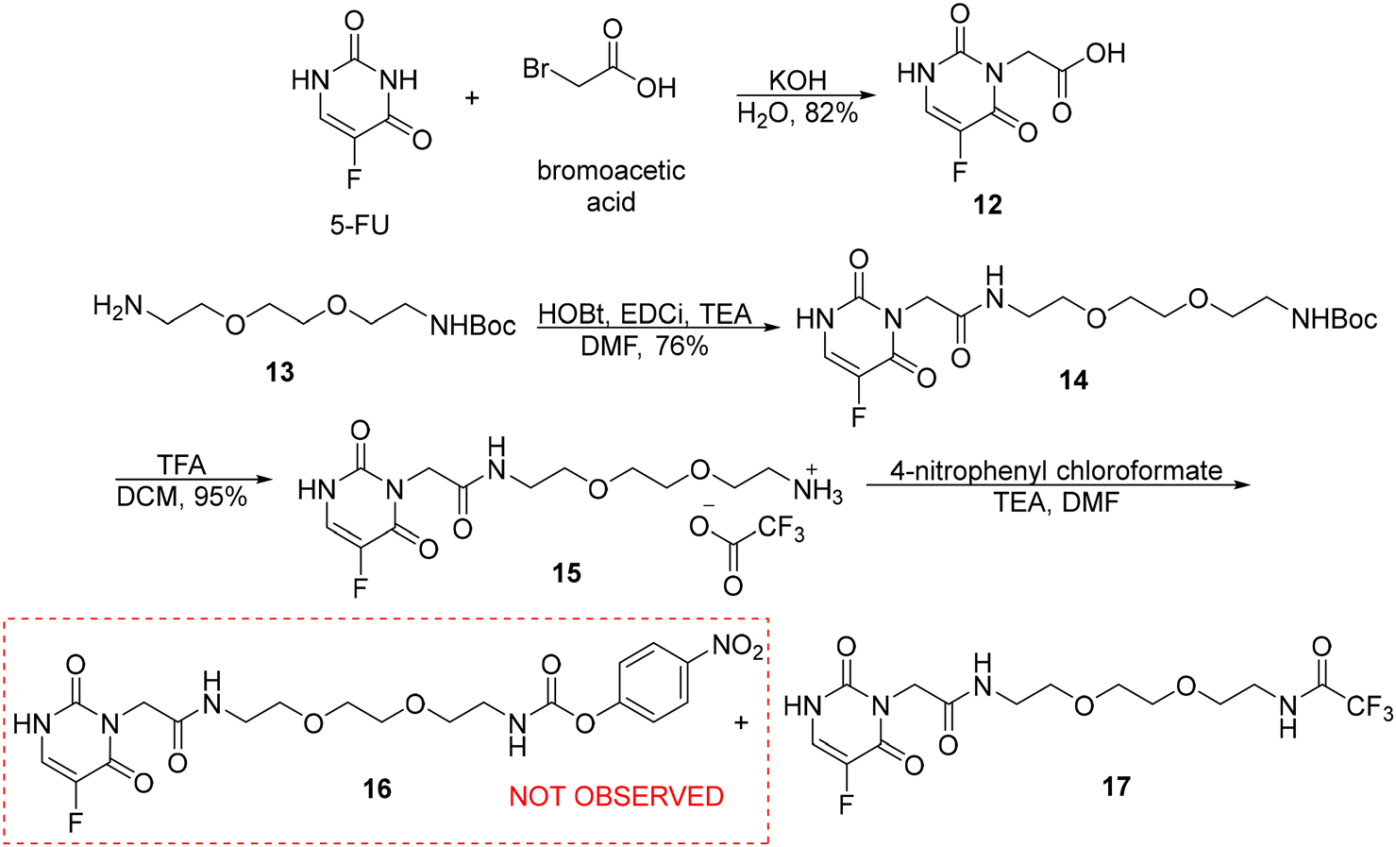
Attempted synthesis of activated ester 16.

The synthesis of the hybrid compound of JH-VII-139-1 with 5-FU, geo140 started with the preparation of the carboxylic acid derivative of the antimetabolite 5-FU, compound 12. As reported in our previous work, 5-FU was alkylated with bromoacetic acid under basic conditions to yield derivative 12 in 82% yield.^13^ Compound 12 was then reacted with the Boc-protected amine 13 in the presence of HOBt, EDCI, and TEA, resulting in the formation of the desired amide 14 in 76% yield (Scheme 3). Compound 14 was deprotected with TFA in DCM, yielding the TFA salt 15 in near-quantitative conversion. In the next step, activation with 4-nitrophenyl chloroformate did not afford the desired activated ester 16, but instead exclusively gave the undesired amide 17. A possible explanation for this result is the nucleophilic attack on the activated ester 16 by trifluoroacetate ions. Despite their weak nucleophilicity, trifluoroacetate ions can react with the highly electrophilic 16 in the presence of a base.

**Scheme 4:**
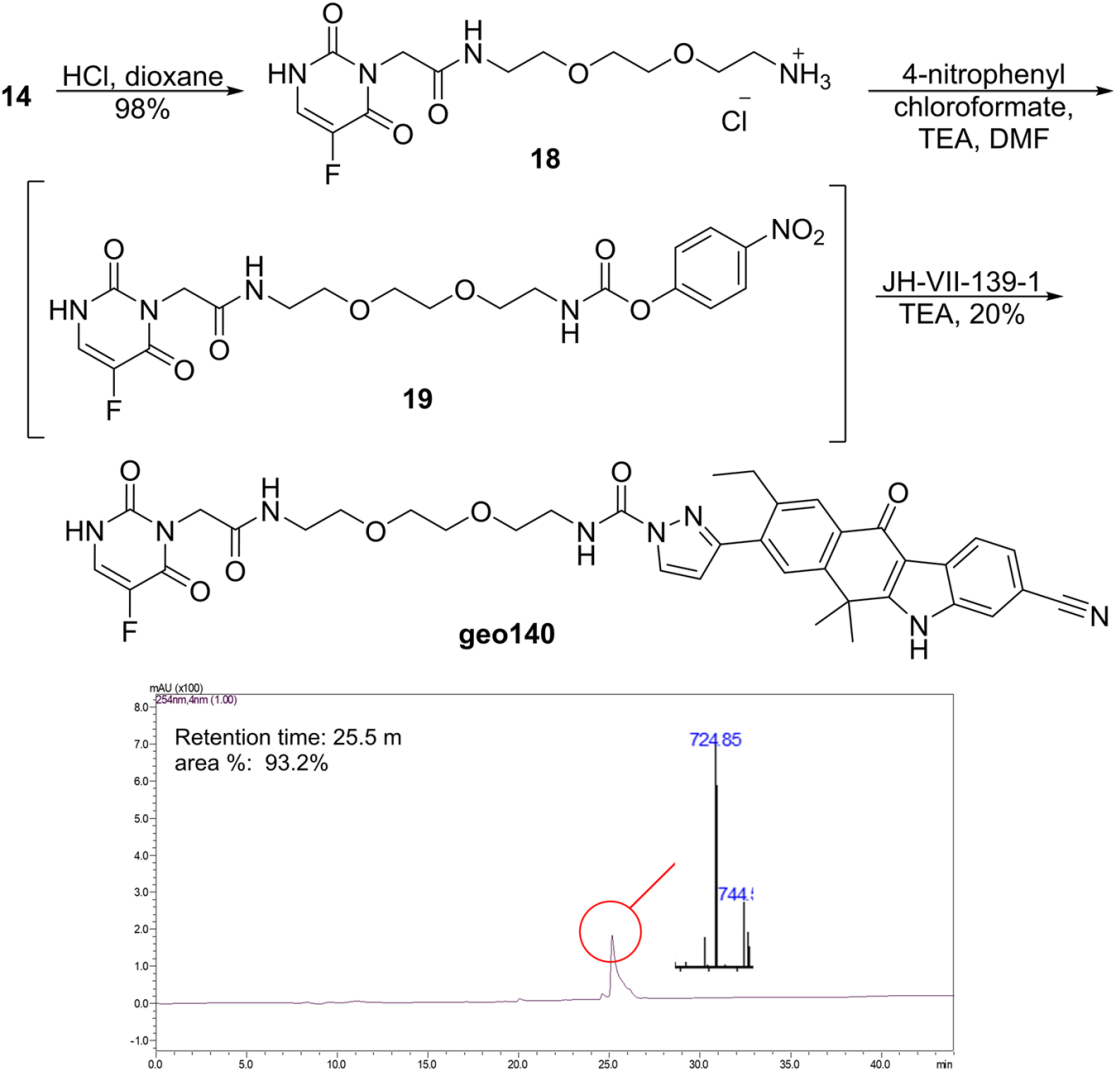
Synthesis of the conjugate geo140 and its LC-MS chromatogram. Method: 1.

The problem was resolved by changing the deprotection conditions of 14. After adding a hydrochloric acid solution in dioxane, the deprotected hydrochloride salt 18 was obtained with a 98% yield (Scheme 4). Activation with 4-nitrophenyl chloroformate ester proceeded easily to the 4-nitrophenyl carbamate 19. Due to its instability, ester 19 was not isolated, and its solution was used in the subsequent step. Finally, the activated ester 19 reacted with the inhibitor JH-VII-139-1 in an amidation reaction in the presence of triethylamine, affording the desired conjugate geo140 in 20% yield. Although quite polar, geo140 was isolated as a green, fluorescent solid after column chromatography with a purity of 93%. The final conjugate has a calculated exact mass for the ion [M + H]^+^ = 725.2842, while a mass of 724.85 was detected in the mass spectrometer.

### *In vitro* stability studies

The stability of SRPK1-targeting conjugates geo15 and geo140 was assessed at pH 5.2 and 7.4, as well as in cell culture medium (DMEM) and human plasma. All experiments were conducted at 37 °C, with samples analysed by LC-ESI-MS at specific time points. Geo140 demonstrated high stability across all tested conditions, as shown in Fig. 4 and S16 and S17. In contrast, geo15 displayed varying stability profiles, with retro-Knoevenagel degradation and the release of 21 being the primary reaction observed. The half-lives of the conjugates ranged from several minutes to several hours, is discussed below.

### Chemostability profile of geo15

**Fig. 3:**
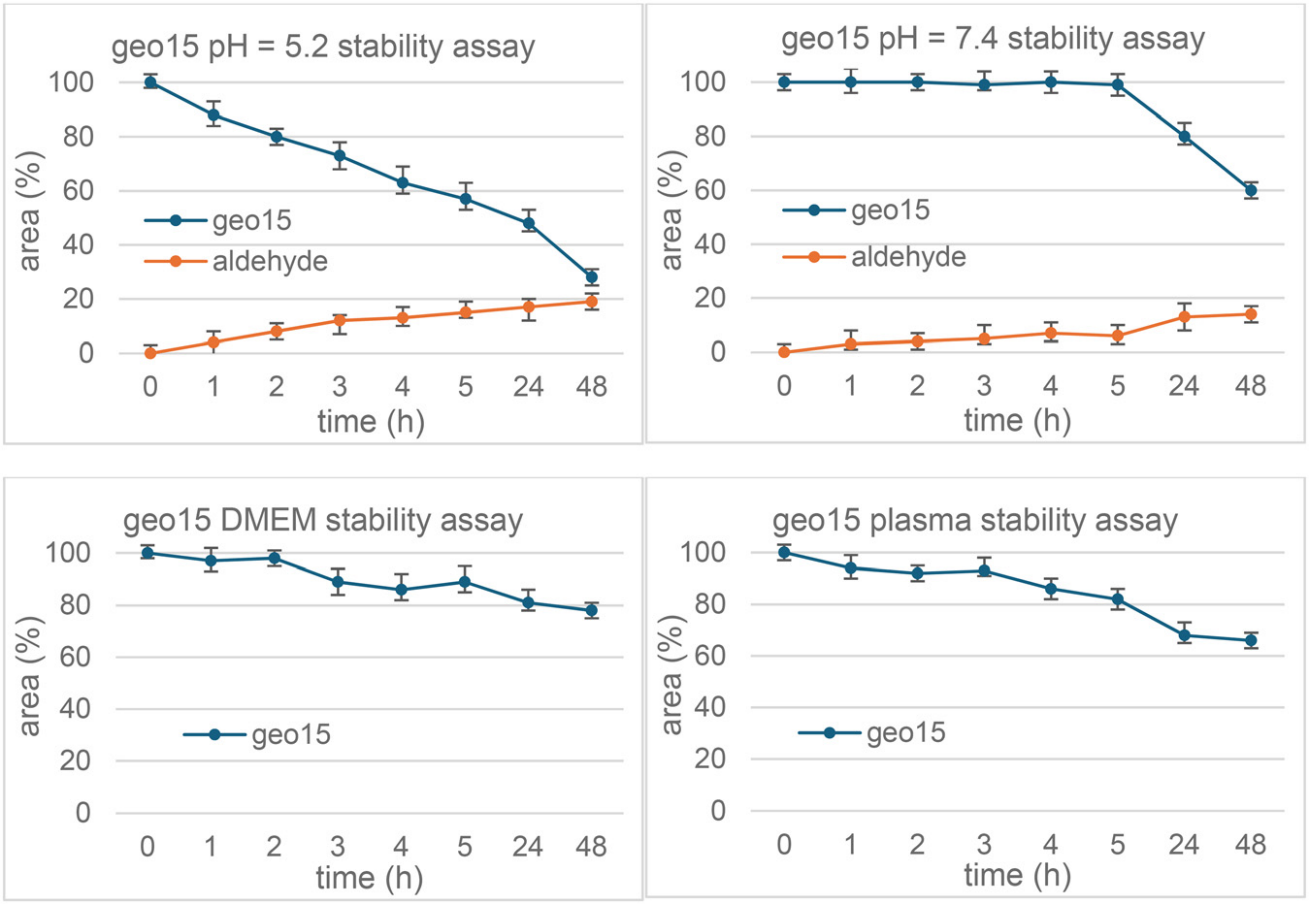
Chemostability of the conjugate geo15.

The relative percentage concentration of geo15 over time is presented, measured at incubation times from *t* = 0 to *t* = 48 (Fig. 3). Similar to the stability of the peptide conjugates of SRPIN803 (geo41 and geo35) published in our previous work,^15^ the hybrid geo15 was less stable in the pH 5.2 buffer (with a half-life of 23 hours and 55 minutes) compared to pH 7.4, DMEM and plasma buffers (half-life greater than 48 hours) (Fig. 3). Nevertheless, the overall stability profile was suitable for further studies, as the concentration of the conjugate remained above 50% during the 24 hour period.

**Scheme 5:**
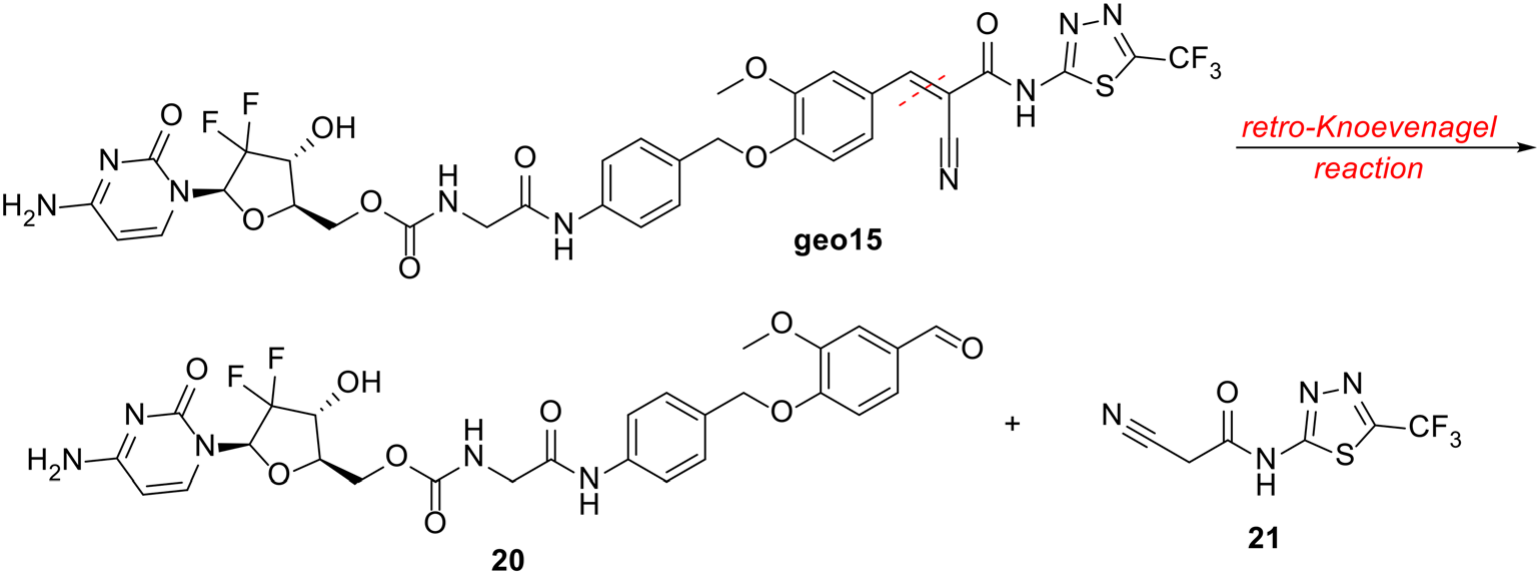
Retro-Knoevenagel reaction of geo15.

A small percentage of the retro-Knoevenagel reaction product 21 (Scheme 5) was also detected in the pH buffer media. However, it was not observed in the DMEM or plasma media, where the conjugate exhibited overall greater stability. Finally, no release of SRPIN803 *via* the 1,6-elimination mechanism was observed. For 1,6-elimination to occur, hydrolysis of the amide bond must first take place, a reaction that may take place inside the cell.

### Chemostability profile of geo140

**Fig. 4:**
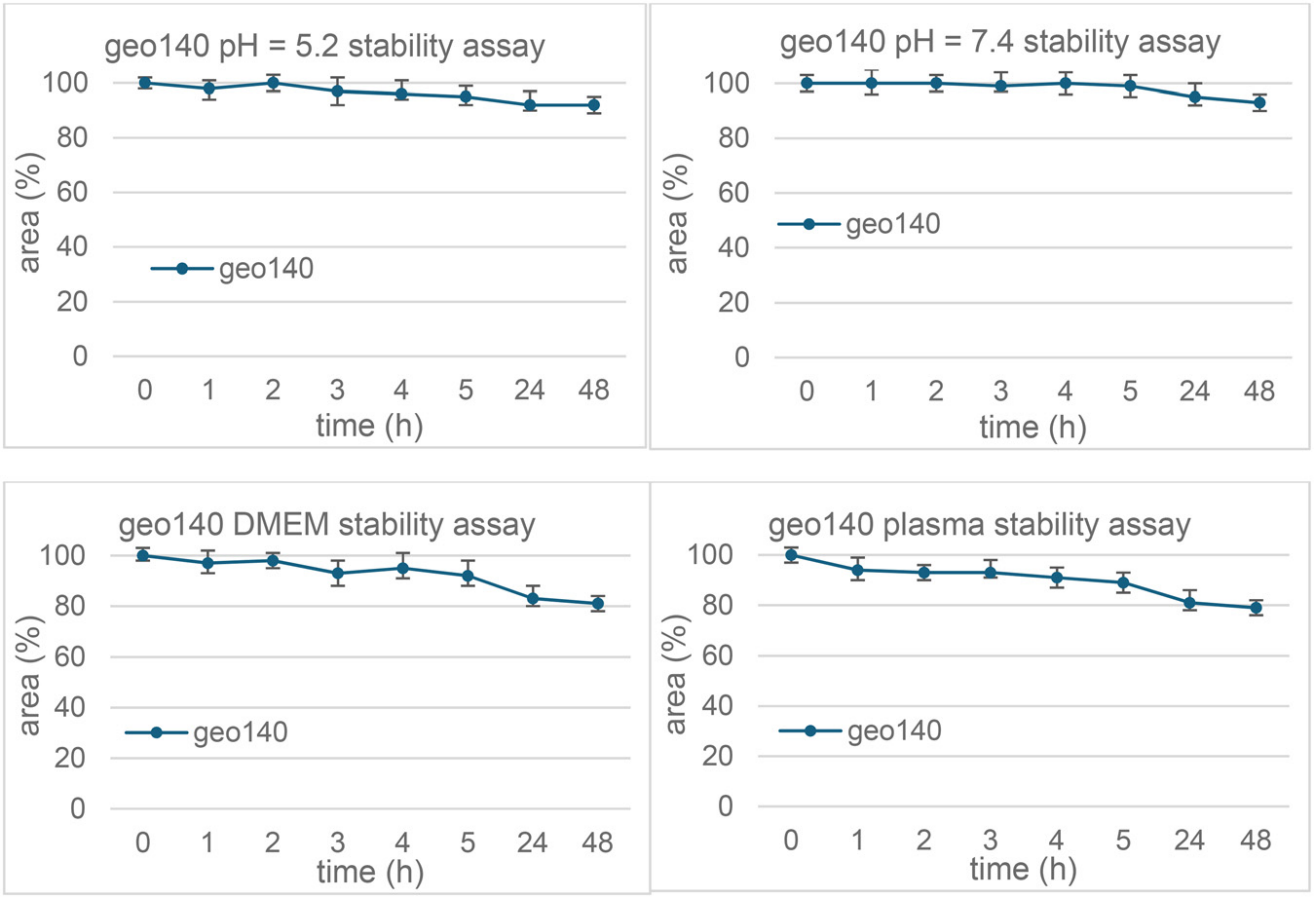
Chemostability of conjugate geo140.

The conjugate geo140 demonstrated great stability in both buffer media, with half-lives exceeding 48 hours. It also remained stable in DMEM and human plasma, although a 10–20% reduction in its initial concentration was observed after 24 hours (Fig. 4). This profile was attributed to the presence of chemically stable amide and urea anchor points.

## Biological activity of the synthesized compounds

### Cytotoxicity of the hybrid compounds geo15 and geo140

To evaluate the cytotoxic effects of the hybrid compounds geo15 and geo140, we performed MTT assays on HeLa, K562, A549, HepG2, HT-29 and Caco2 cancer cell lines, as well as primary intestinal subepithelial myofibroblasts (SEMFs). The half-maximal effective concentration (EC_50_) values were determined after 48 hours of treatment and are presented in Tables 1 and 2.

**Table 1:** Half maximal effective concentrations (EC_50_ values) of 5-FU, JH-VII-139-1 and geo140, in cancer cell lines HeLa, K562, A549, HepG2, HT-29, Caco2 and SEMFs following treatment for 48 h. Data represent the mean of three independent experiments

	EC_50_ (μM)						
	HeLa	K562	A549	HepG2	HT-29	Caco2	SEMFs
5-FU	25.7	>50	15.19	>50	3.62	>50	>50
JH-VII-139-1	8.6	13.2	>50	16.66	1.4	14.77	12.84
geo140	11.7	13.7	>50	27.14	19.33	31.24	21.5

**Table 2:** Half maximal effective concentrations (EC_50_ values) of gemcitabine, SRPIN803 and geo15, in cancer cell lines HeLa, K562, A549, HepG2, HT-29, Caco2 and SEMFs following treatment for 48 h. The data represent the mean of three independent experiments

	EC_50_ (μM)						
	HeLa	K562	A549	HepG2	HT-29	Caco2	SEMFs
Gemcitabine	6.7	>50	>50	31.44	28.67	>50	>50
SRPIN803	60.1	>50	14.45	32.51	>50	31.64	>50
geo15	33.1	>50	>50	>50	38.36	>50	>50

Geo140, the hybrid compound of 5-FU with JH-VII-139-1, exhibited significant cytotoxic activity across all cell lines tested, apart from A549 cells. The EC_50_ values for geo140 for every cell line are listed in Table 1. In all cell lines tested geo140 cytotoxic effect was less potent than its parent compound JH-VII-139-1, with HeLa and K562 being the most sensitive cell lines.

Geo15, the hybrid of gemcitabine and SRPIN803, presented reduced cytotoxicity in all cell lines tested as compared to its parent compounds, except for HeLa and HT-29 cells, in which geo15 was more potent than SRPIN803.

Given the limited cytotoxicity of geo15 and its lack of efficacy in K562, A549, HepG2, Caco2 cells and primary SEMFs, we decided not to extend its biological evaluation further and continued our studies with geo140 which demonstrated a more consistent cytotoxic effect towards almost all the tested cell lines.

### Inhibition of SRPK1 and SRPK2 kinase activity by JH-VII-139-1 and the hybrid compound geo140

**Fig. 5:**
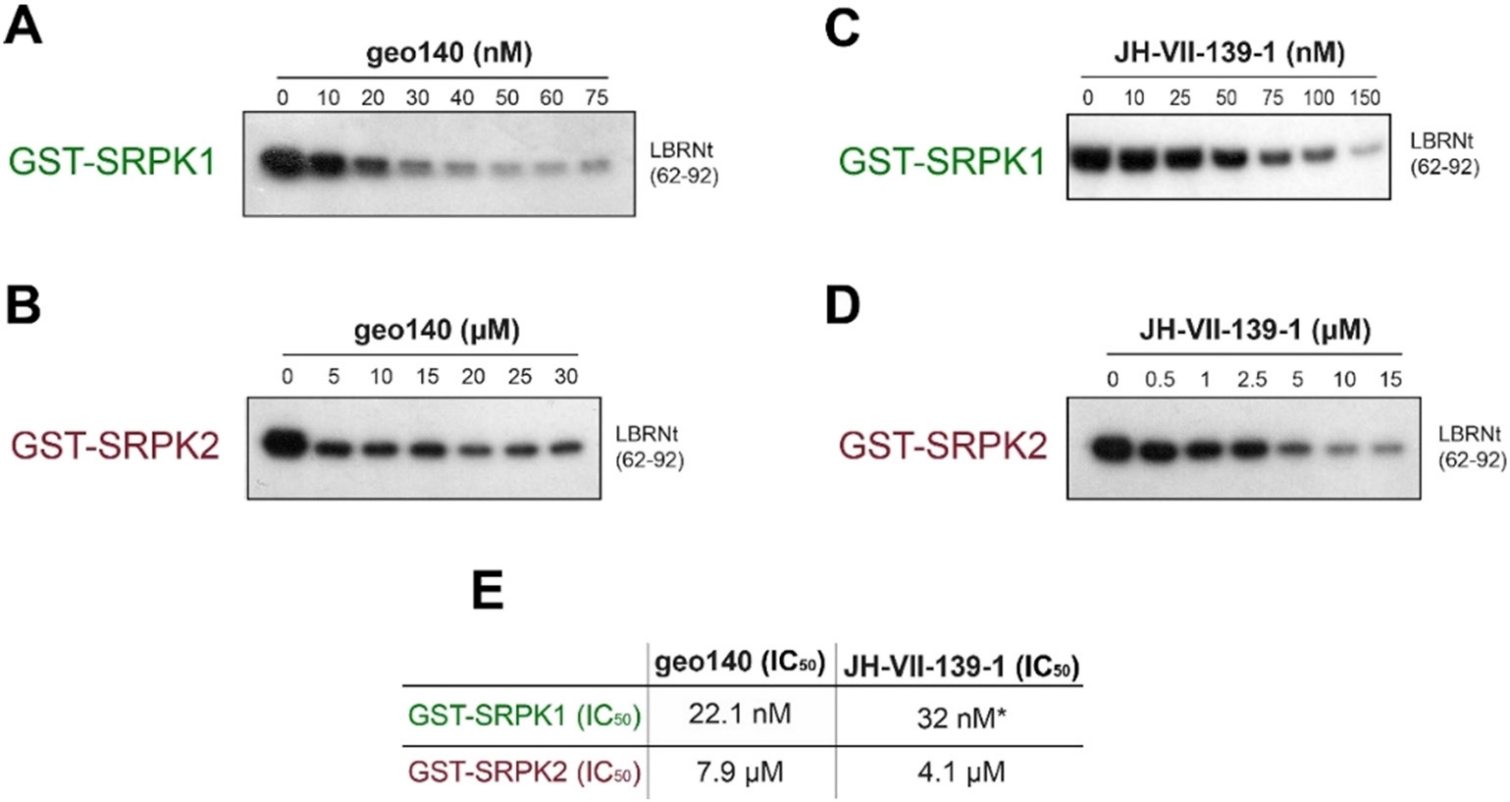
Phosphorylation of 1 μg GST-LBRNt(62–92) by 0.5 μg GST-SRPK1 or 0.5 μg GST-SRPK2 in the presence of increasing concentrations of geo140 (A and B, respectively) or in the presence of increasing concentrations of JH-VII-139-1 (C and D, respectively). Panel C was taken from a previous publication of the authors.^2^ The samples were analyzed by SDS-PAGE on 12% gels, stained with Coomassie blue and autoradiographed. Only the relevant part of the autorad corresponding to the phosphorylated substrate is shown. E. IC_50_ values of geo140 and JH-VII-139-1 against GST-SRPK1 and GST-SRPK2.

The inhibitory activity of geo140 and JH-VII-139-1 against SRPK1 and SRPK2 was assessed using *in vitro* kinase assays (Fig. 5). Geo140 inhibited the activity of SRPK1 and SRPK2 toward LBRNt(62–92), a well-known substrate of the kinase^20^ with IC_50_ values of 22.1 nM and 7.9 μM, respectively, comparable with the respective IC_50_ values obtained with JH-VII-139-1 alone (Fig. 5E). The IC_50_ values summarized in Panel E indicate that both JH-VII-139-1 and the hybrid compound geo140 act as effective inhibitors of SRPK-mediated phosphorylation, with however, differential selectivity between SRPK1 and SRPK2.

### Effect of geo140 on the subcellular localization of SRPK1

In a following step the effect of geo140 was tested on the subcellular distribution of SRPK1 in HeLa cells. To address this issue, a series of immunofluorescence experiments was performed to assess the effect of geo140, relative to its parent compounds, JH-VII-139-1 and 5-FU on SRPK1 localization.

**Fig. 6:**
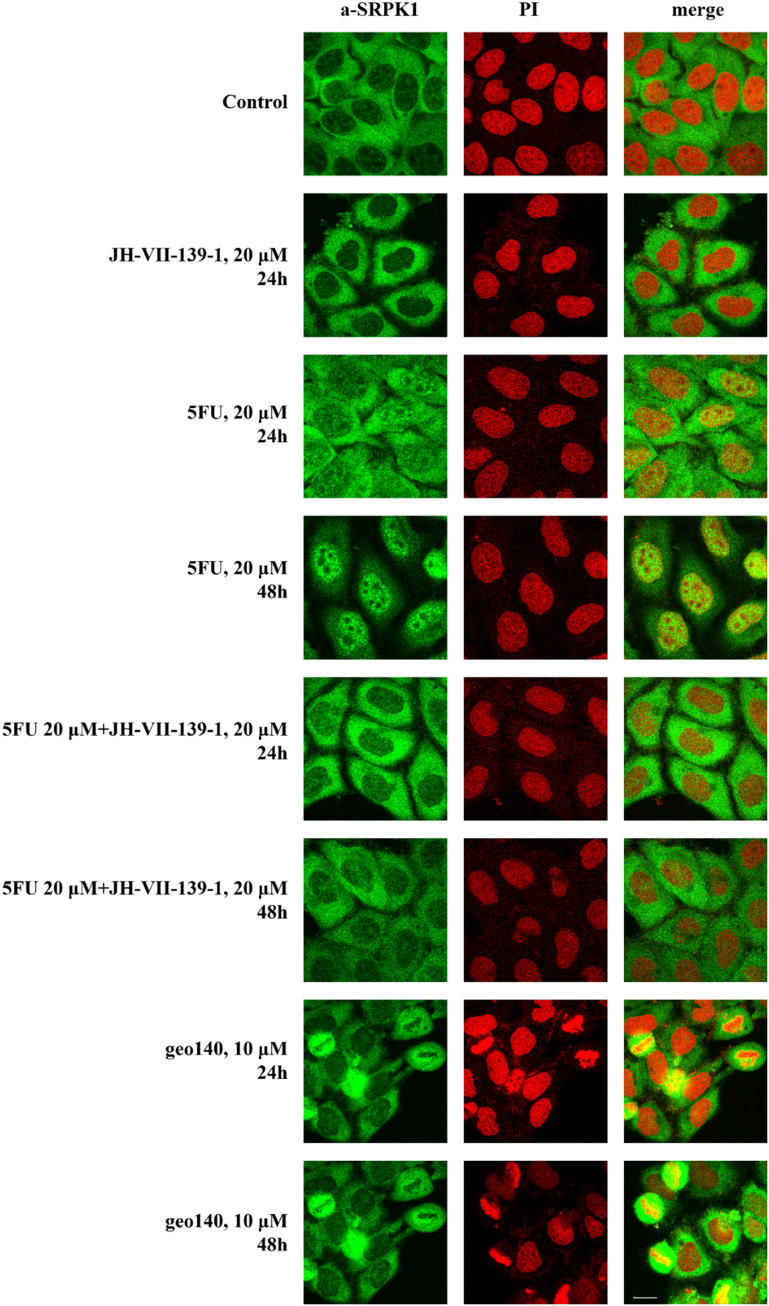
Fluorescent patterns of SRPK1 in HeLa cells treated with JH-VII-139-1, 5-FU, and geo140 (for the indicated concentrations and time periods). SRPK1 was detected using an anti-SRPK1 monoclonal antibody (Alexa488-green fluorescence) while nuclei were stained with PI (red fluorescence). Scale bar: 10 μm.

In control cells, SRPK1 predominantly localized to cytoplasm, while treatment with JH-VII-139-1 (20 μM, 24 h) did not affect the cytoplasmic localization of the kinase (Fig. 6). Exposure of cells to 20 μM 5-FU for 24 h, led to a more diffuse SRPK1 staining pattern, with some nuclear translocation of the kinase, while treatment of cells with 5-FU, for 48 h, resulted in almost complete nuclear translocation of SRPK1, corroborating previous reports showing the induction of SRPKs' nuclear translocation in response to stress and especially chemotherapeutic agents.^21–24^ As the kinase activity is essential for the entry of SRPKs into the nucleus, addition of the inhibitor JH-VII-139-1, prevented the nuclear accumulation of SRPK1 in 5-FU-treated cells, in accordance with previous observations.^16^

Interestingly, addition of the newly synthesized hybrid compound, geo140 resulted in a different pattern than that observed with co-treatment of cells with JH-VII-139-1 and 5-FU. While in interphase cells SRPK1 retained a cytoplasmic distribution similar to that seen in untreated controls, a significant increase in the number of mitotic cells was observed. In these cells, SRPK1 was not associated but rather surrounded by condensed chromosomes.

**Fig. 7:**
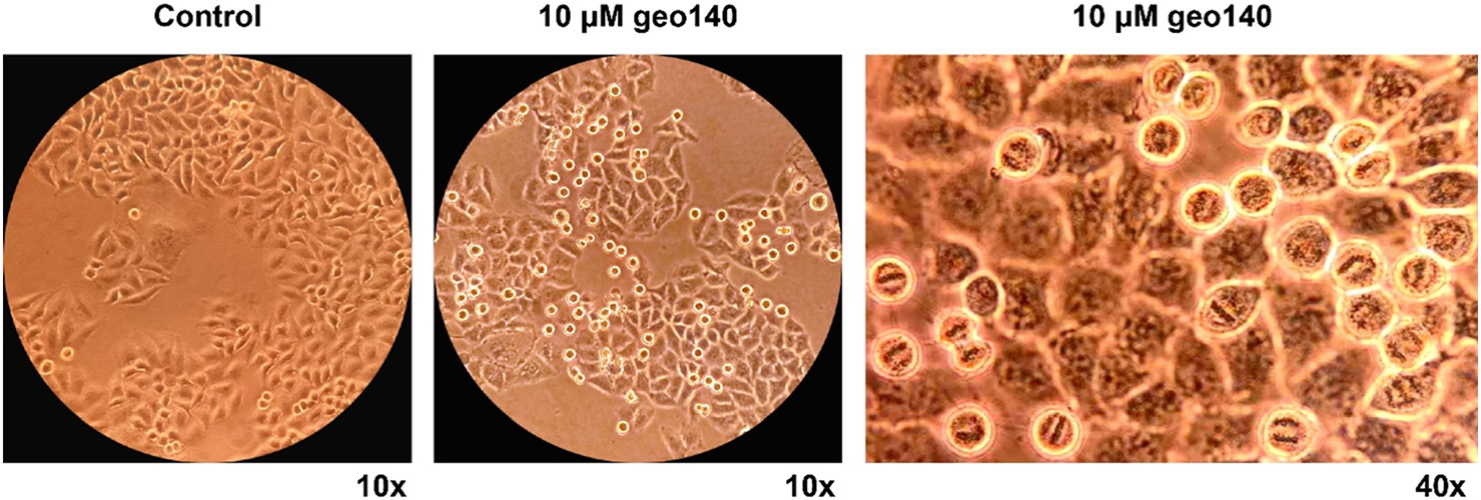
Microscopic view of control HeLa cells and treated with 10 μM geo140, taken at 10× or 40× magnification as indicated, using an inverted phase-contrast microscope.

A similar morphology of geo140-treated cells was also observed using an inverted phase-contrast microscope. As shown in Fig. 7, a significant increase in mitotic cells, characterized by their round shape and distinct chromosomal structures, was observed.

These observations suggest that a significant number of HeLa cells are unable to exit mitosis upon treatment with geo140. Further studies are required to clarify the mechanism of geo140 imposing a mitotic arrest and the potential involvement of SRPK1 on it.

## Conclusion

In this work, we aimed to develop dual-targeting conjugates capable of simultaneously inhibiting the SRPK1 kinase while incorporating antimetabolite functionality to achieve synergistic anticancer effects. Building on the experiences gained from our work with peptide drug conjugates, we were inspired by previous studies showing that targeting of SRPK1 results in enhanced sensitivity to platinum-based chemotherapy in some types of cancer.^2,4^ Two hybrid molecules, geo15 and geo140, were designed and synthesized which combine known SRPK1 inhibitors (SRPIN803, JH-VII-139-1) with the antimetabolites gemcitabine and 5-FU. The hybrid of 5-FU with JH-VII-139-1, geo140, exhibited significant cytotoxic activity comparable to JH-VII-139-1 across all cell lines tested except A549. On the other hand, the hybrid of gemcitabine with SRPIN803, geo15, presented reduced cytotoxicity in all cell lines tested as compared to its parent compounds, except for HeLa and HT-29 cells, in which geo15 was more potent than SRPIN803. The conjugate geo140 preserved the activity of the parent compound JH-VII-139-1 against SRPK1 and SRPK2 with IC_50_ values of 22.1 nM and 7.9 μM, respectively. Therefore, we noticed that the antiproliferative efficacy of the conjugates geo140 and geo15 was correlated with the activity of the SRPK1 inhibitor. JH-VII-139-1 is a stable and nanomolar SRPK1 inhibitor, whereas SRPIN803 is a micromolar inhibitor. Although 5-FU exhibits lower cytotoxicity than gemcitabine, its conjugation with JH-VII-139-1 yielded a hybrid compound with enhanced cytotoxic potency particularly against HeLa and K562 cells. When examining the effect of geo140 on the subcellular localization of SRPK1 we found that the conjugate geo140 resulted in a different phenotype than that observed with co-treatment of cells with JH-VII-139-1 and 5-FU. Our results demonstrate that compound geo140 did not alter the subcellular localization of SRPK1, but had a profound effect on cell cycle regulation. This observation may be explained by a novel mechanism of action of the hybrid, involving the dual activity against SRPK1 and antimetabolite-mediated inhibition of cell growth. Alternatively, geo140 may exert its effects by interfering with a different molecular target. While additional studies are warranted to elucidate the molecular mechanisms underlying these observations, this work supports the potential of molecular hybridization as an innovative approach for the development of novel SRPK1-targeting anticancer agents with potential therapeutic relevance.

## Chemistry

All reactions were conducted under an argon atmosphere unless otherwise specified, using commercial reagents without additional purification. Reactions were monitored by thin-layer chromatography (TLC), visualized under UV light, and developed with aqueous ceric sulfate/phosphomolybdic acid, ethanolic *p*-anisaldehyde solution, potassium permanganate solution, and heat. ^1^H and ^13^C NMR spectra were recorded at 500 MHz and 126 MHz, respectively, on an Agilent spectrometer, with tetramethylsilane (TMS) as an internal standard. Chemical shifts are reported in *δ* (ppm) from internal reference peaks (TMS ^1^H 0.00; CDCl_3_^1^H 7.26, ^13^C 77.16, (CD_3_)_2_SO ^1^H 2.50, ^13^C 39.52, (CD_3_)_2_CO ^1^H 2.05, ^13^C 29.84, 206.26). LC-MS analysis was performed on a Shimadzu LC-20 AD system connected to a Shimadzu LCMS-2010EV (Shimadzu, Kyoto, Japan), equipped with a C_18_ analytical column (Supelco Discovery C18, 5 μm, 250 × 4.6 mm).

### (*tert*-Butoxycarbonyl)glycine, 4

In a solution of glycine (1.00 g, 13.3 mmol) and saturated potassium hydroxide (29 g, 520 mmol) in dioxane and water (40 mL, 1 : 1), Boc_2_O (3.7 mL, 16.0 mmol) was added. The addition was carried out at room temperature, and the reaction was stirred for 12 hours. After the reaction was complete, the solvent was evaporated, and the residue was dissolved in ethyl acetate. The organic phase was washed with 10% aqueous sodium sulfate and brine. The organic layers were collected, dried over Na_2_SO_4_, and concentrated *in vacuo*. The white solid residue 4 (2.33 g, 43%) was used in subsequent reactions without any further purification. The spectroscopic data agree with the literature values.^25^4: ^1^H NMR (500 MHz, CDCl_3_) *δ* 5.04 (s, 1H), 3.96 (s, 2H), 1.45 (s, 9H).

### 2,5-Dioxopyrrolidin-1-yl(*tert*-butoxycarbonyl)glycinate, 5

To a solution of Boc-glycine 4 (141 mg, 0.805 mmol) and NHS (140 mg, 1.22 mmol) in dry THF (2 mL), a solution of DCC (327 mg, 1.95 mmol) in dry THF (2 mL) was added. The addition takes place under an argon atmosphere, and the reaction was stirred for 24 hours at room temperature. During the work-up, a few drops of acetic acid were added, and after 1 hour of stirring, the mixture was filtered under vacuum to remove the insoluble DCU. The filtrate was then concentrated, and the resulting residue was dissolved in 2-propanol and stirred at room temperature for 1 hour. The resulting suspension was filtered under vacuum and washed with 2-propanol. Finally, 5 (132 mg, 60%) was obtained as a white solid and used in subsequent reactions without further purification.^26^5: ^1^H NMR (500 MHz, CDCl_3_) *δ* 4.95 (s, 1H), 4.29 (d, *J* = 5.4 Hz, 2H), 2.85 (s, 4H), 1.46 (s, 9H).

### *tert*-Butyl (2-((4-(hydroxymethyl)phenyl)amino)-2-oxoethyl)carbamate, 6

A solution of Boc-glycine *N*-hydroxysuccinimide ester 5 (110 mg, 0.404 mmol) and *para*-aminobenzyl alcohol (100 mg, 0.812 mmol) in dry dichloromethane (1.5 mL) was stirred under an argon atmosphere at room temperature for 48 hours. The reaction was concentrated, and the resulting residue was redissolved in dichloromethane and washed with a 0.1 N hydrochloric acid solution (0.5 mL) and water (0.5 mL). The organic layers were collected, dried over Na_2_SO_4_, and concentrated under vacuum. The solid residue was subjected to column chromatography (petroleum ether/ethyl acetate, gradient elution from 1/1 to 1/2), and the desired alcohol 6 (80 mg, 71%) was obtained as a brown oil. Spectroscopic data were in agreement with the literature values.^27^^1^H NMR (500 MHz, CDCl_3_) *δ* 8.08 (s, 1H), 7.51 (d, *J* = 8.3 Hz, 2H), 7.33 (d, *J* = 8.3 Hz, 2H), 5.17 (s, 1H), 4.66 (s, 2H), 3.92 (d, *J* = 6.0 Hz, 2H), 1.49 (s, 9H).

### *tert*-Butyl (2-((4-(bromomethyl)phenyl)amino)-2-oxoethyl)carbamate, 7

To a cold (0 °C) solution of 6 (67 mg, 0.239 mmol) in dry dichloromethane (3.3 mL), phosphorus tribromide (7.64 μL, 0.081 mmol) was added. The addition was made under an argon atmosphere, and the reaction was stirred for 30 minutes at 0 °C. After that, cold water was added to the reaction, followed by extractions with dichloromethane. The organic layers were collected, dried over Na_2_SO_4_, and concentrated in vacuum. The obtained yellow solid 7 (37 mg, 45%) was used in subsequent reactions without any further purification. 7: ^1^H NMR (500 MHz, CDCl_3_) *δ* 8.14 (s,1H), 7.49 (d, *J* = 8.5 Hz, 2H), 7.35 (d, *J* = 8.5 Hz, 2H), 5.17–5.11 (m,1H), 4.48 (s, 2H), 3.91 (d, *J* = 6.1 Hz, 2H), 1.53 (s, 9H).

### *tert*-Butyl (*E*)-(2-((4-((5-(2-cyano-3-oxo-3-((5-(trifluoromethyl)-1,3,4-thiadiazol-2-yl)amino)prop-1-en-1-yl)-2-methoxyphenoxy)methyl)phenyl)amino)-2-oxoethyl)carbamate, 8

To a solution of 7 (177 mg, 0.516 mmol) and SRPIN803 (210 mg, 0.567 mmol) in dry dimethylformamide (3.83 mL), potassium carbonate (78.4 mg, 0.567 mmol) were added. The addition was made at room temperature, and the reaction was left stirring for 24 hours at room temperature. The solvent was removed *in vacuo*, and the resulting yellow residue 8 (426 mg, 46%) was used in subsequent reactions without any further purification. 8: ^1^H NMR (500 MHz, DMSO-*d*_6_) *δ* 10.01 (s, 1H), 7.74 (d, *J* = 8.5 Hz, 1H), 7.56–7.52 (m, 3H), 7.42 (d, *J* = 8.5 Hz, 1H), 7.38 (d, *J* = 8.5 Hz, 2H), 7.05–7.02 (m, 1H), 5.40 (s, 1H), 4.68 (s, 2H), 3.71 (d, *J* = 5.9 Hz, 2H), 1.39 (s, 9H), –OCH_3_ peak is overlapping with DMSO-*d*_6_ water peak. ^13^C NMR (126 MHz, DMSO-d_6_) *δ* 169.1, 168.8, 165.4, 163.9, 156.4, 141.4, 139.4, 138.0, 133.0, 132.8, 130.4, 127.4, 122.3, 120.0, 119.5, 119.2, 117.9, 110.7, 105.2, 78.5, 63.0, 44.2, 35.3, 28.7. ESI-MS, positive mode: *m*/*z* calcd mass for C_28_H_27_F_3_N_6_O_6_S [M + H]^+^ = 633.1738, was found 633.15.

### (*E*)-3-(3-((4-(2-Aminoacetamido)benzyl)oxy)-4-methoxyphenyl)-2-cyano-*N*-(5-(trifluoromethyl)-1,3,4-thiadiazol-2-yl)acrylamide TFA salt, 9

To a solution of 8 (326 mg, 0.515 mmol) in dry dichloromethane (4.8 mL), a solution of trifluoroacetic acid in dichloromethane (2.4 mL, 31.4 mmol in 2.4 mL) was added dropwise. The addition was made under argon atmosphere, at 0 °C, and the reaction was stirred for 20 minutes at 0 °C. After the formation of TFA salt, the reaction mixture was concentrated under reduced pressure, and the resulting residue was purified by preparative thin-layer chromatography (dichloromethane/methanol 10 : 2), to yield the desired compound 9 (128 mg, 47%) as a yellow, fluorescent solid. 9: ^1^H NMR (500 MHz, DMSO-*d*_6_) *δ* 8.36 (s, 1H), 7.78 (d, *J* = 1.8 Hz, 1H), 7.63 (d, *J* = 8.5 Hz, 2H), 7.59 (dd, *J* = 9.0, 1.7 Hz, 1H), 7.48 (d, *J* = 8.5 Hz, 2H), 6.80 (d, *J* = 8.5 Hz, 1H), 5.63 (s, 2H), 3.80 (s, 3H), 3.48 (s, 2H). ^13^C NMR (126 MHz, DMSO-*d*_6_) *δ* 170.8, 168.9, 164.1, 152.8 (*J* = 4.2 Hz), 148.6, 144.7 (*J* = 40.9 Hz), 138.7, 129.6, 129.4, 129.3, 129.2, 128.9, 122.4, 121.1, 119.2, 119.1, 119.0, 118.5, 117.1, 113.1, 109.5, 55.4, 54.4, 43.4. ESI-MS, positive mode: *m*/*z* calcd mass for C_23_H_19_F_3_N_6_O_4_S [M + H]^+^ = 533.1213, was found 533.00.

### Boc-protected SRPIN803-gem conjugate, 11

To a solution of compound 9 (5 mg, 0.0105 mmol) and compound 10 (6.61 mg, 0.0105 mmol) in dry dimethylformamide (0.176 mL), dry triethylamine (2.93 μL, 0.021 mmol) was added. The addition was performed under an argon atmosphere, and the reaction was stirred for 2 days at room temperature. The solvent was removed under vacuum, and the resulting residue was subjected to column chromatography (petroleum ether/ethyl acetate, gradient elution from 1 : 1 to 1 : 5) to isolate the desired protected conjugate 11 (11 mg, 67%) as a fluorescent yellow solid. ^1^H NMR (500 MHz, acetone-*d*_6_) *δ* 9.35 (s, 1H), 8.51 (s, 1H), 8.04 (d, *J* = 7.6 Hz, 1H), 7.95 (d, *J* = 1.9 Hz, 1H), 7.71–7.68 (m, 3H), 7.58 (d, *J* = 8.4 Hz, 2H), 7.32 (d, *J* = 7.7 Hz, 1H), 7.04 (d, *J* = 8.3 Hz, 1H), 6.96 (s, 1H), 6.38 (s, 1H), 5.72–5.70 (m, 2H), 5.34–5.28 (m, 1H), 4.64 (d, *J* = 12.2 Hz, 1H), 4.48–4.43 (m, 1H), 4.39 (dd, *J* = 12.5, 4.0 Hz, 1H), 4.05–3.97 (m, 2H), 3.95 (s, 3H), 1.51 (s, 9H), 1.48 (s, 9H). ^13^C NMR (126 MHz, acetone-*d*_6_) *δ* 171.6, 168.5, 166.1, 164.6, 157.1, 155.0, 154.4, 153.1, 152.7, 152.5, 146.7, 145.8, 140.2, 130.8, 130.3, 128.6, 125.2, 123.3, 120.4, 119.7, 118.2, 116.6, 114.0, 105.5, 96.1, 84.5, 82.5, 78.3, 73.8, 62.8, 56.4, 55.8, 45.5, 45.3, 28.2, 27.7. ESI-MS, positive mode: *m*/*z* calcd mass for C_43_H_44_F_5_N_9_O_13_S [M + H]^+^ = 1022.2772, was found 1021.95.

### Conjugate geo15

In a cold solution of 11 (3.8 mg, 3.7 μmol) in dry dichloromethane (60 μL), a solution of trifluoroacetic acid (17.4 μL, 0.227 mmol) in dichloromethane (30 μL) was added dropwise. The addition was performed under an argon atmosphere, and the reaction was stirred at 0 °C for 30 minutes. The solvent was removed by lyophilization, and the crude residue was subjected to semi-preparative HPLC (method 3) to isolate geo15 (1.5 mg, 90%) as a fluorescent yellow solid. ^1^H NMR (500 MHz, DMSO-*d*_6_) *δ* 10.07 (s, 1H), 8.47 (s, 1H), 7.83 (s, 1H), 7.70–7.64 (m, 2H), 7.60 (d, *J* = 8.4 Hz, 2H), 7.52 (d, *J* = 7.5 Hz, 1H), 7.47 (d, *J* = 8.4 Hz, 2H), 7.43–7.35 (m, 2H), 6.94 (d, *J* = 8.3 Hz, 1H), 6.42 (s, 1H), 6.22–6.17 (m, 1H), 5.82 (d, *J* = 7.5 Hz, 1H), 5.65 (s, 2H), 4.37 (d, *J* = 11.4 Hz, 1H), 4.24–4.17 (m, 2H), 3.96 (s, 1H), 3.84 (s, 3H), 3.82–3.79 (m, 2H). ^13^C NMR (126 MHz, DMSO-*d*_6_) *δ* 170.5, 168.0, 167.7165.6, 164.7, 156.2, 154.6, 153.6, 150.4, 148.8, 148.1, 145.0, 141.1, 139.0, 129.5, 129.1, 127.8, 122.8, 121.3, 121.1, 119.2, 118.4, 117.8, 116.3, 113.7, 102.4, 95.0, 77.5, 69.8, 62.3, 55.6, 54.5, 44.0. ESI-MS, positive mode: *m*/*z* calcd mass for C_33_H_28_F_5_N_9_O_9_S [M + H]^+^ = 822.1724, was found 822.0.

### *tert*-Butyl (2-(2-(2-(2-(5-fluoro-2,6-dioxo-3,6-dihydropyrimidin-1(2*H*)-yl)acetamido)ethoxy)ethoxy)ethyl)carbamate, 14

To a solution of carboxylic acid derivative 12 (67 mg, 0.356 mmol) and amine 13 (88.4 mg, 0.356 mmol) in dry dimethylformamide (5 mL), HOBt (72.2 mg, 0.534 mmol), triethylamine (150 μL, 1.07 mmol), and EDCi (102 mg, 0.534 mmol) were added. The additions were performed under an argon atmosphere, and the reaction was stirred for 24 hours at room temperature. After completion, the solvent was removed under vacuum, and the resulting residue was subjected to column chromatography (dichloromethane/methanol, gradient elution from 10/0.3 to 10/1.5) to isolate the desired amide 14 (114 mg, 76%) as a white crystalline solid. ^1^H NMR (500 MHz, acetone-*d*_6_) *δ* 7.82 (d, *J* = 6.5 Hz, 1H), 7.54 (s, 1H), 5.93 (s, 1H), 4.45 (s, 2H), 3.57 (s, 4H), 3.54–3.46 (m, 4H), 3.39 (q, *J* = 5.5 Hz, 2H), 3.22 (q, *J* = 5.8 Hz, 2H), 1.40 (s, 9H). ^13^C NMR (126 MHz, acetone-*d*_6_) *δ* 167.4, 158.1, 156.7, 150.6, 140.7, 131.3, 78.7, 71.0, 70.8, 70.6, 70.2, 50.6, 41.0, 40.1, 28.6. ESI-MS, negative mode: *m*/*z* calcd mass for C_17_H_27_FN_4_O_7_ [M–H]^−^ = 417.1791, was found 418.00.

### *N*-(2-(2-(2-Aminoethoxy)ethoxy)ethyl)-2-(5-fluoro-2,6-dioxo-3,6-dihydropyrimidin-1(2*H*)-yl)acetamide TFA salt, 15

To a solution of compound 14 (94 mg, 0.224 mmol) in dry dichloromethane (2 mL), a solution of trifluoroacetic acid in dichloromethane (1 mL, 13.7 mmol in 1 mL) was added dropwise. The addition was performed under argon atmosphere at 0 °C, and the reaction was stirred for 20 minutes at 0 °C. The solvent was evaporated under vacuum, and the residue was washed with hexane/dichloromethane (1 : 1) and then with dichloromethane. After the washes, the resulting salt 15 (95 mg, 95%) was collected as a white solid and used in subsequent reactions without any further purification. 15: ESI-MS, positive mode: *m*/*z* calcd mass for C_12_H_19_FN_4_O_5_ [M + H]^+^ = 319.1412, was found 318.80.

### 2,2,2-Trifluoro-*N*-(2-(2-(2-(2-(5-fluoro-2,6-dioxo-3,6-dihydropyrimidin-1(2*H*)-yl)acetamido)ethoxy)ethoxy)ethyl)acetamide, 17

To a solution of compound 15 (95 mg, 0.22 mmol) in dry dimethylformamide (4.06 mL) and triethylamine (111 mg, 1.10 mmol), 4-nitrophenyl chloroformate (66.4 mg, 0.33 mmol) was added and the reaction turns yellow in colour. The addition was performed under an argon atmosphere at 0 °C, and the reaction was stirred for 3 hours at room temperature. The reaction mixture was filtered under vacuum and washed with ethyl acetate. The filtrate was concentrated under vacuum, and the residue was subjected to column chromatography (dichloromethane/methanol, isocratic elution 10 : 1) to obtain the carbamate analogue 239 (38 mg, 42%) as a white solid. 17: ^1^H NMR (500 MHz, acetone-*d*_6_) *δ* 8.52 (s, 1H), 7.81 (d, *J* = 6.5 Hz, 1H), 7.56 (s, 1H), 4.44 (s, 2H), 3.64–3.60 (m, 2H), 3.60–3.55 (m, 4H), 3.54–3.49 (m, 4H), 3.43–3.37 (m, 2H). ESI-MS, positive mode: *m*/*z* calcd mass for C_14_H_18_F_4_N_4_O_6_ [M + H]^+^ = 413.1090, was found 412.95.

### *N*-(2-(2-(2-Aminoethoxy)ethoxy)ethyl)-2-(5-fluoro-2,6-dioxo-3,6-dihydropyrimidin-1(2*H*)-yl)acetamide hydrochloride, 18

To a solution of compound 14 (36 mg, 0.086 mmol) in dry dioxane (0.39 mL), a solution of hydrochloric acid in dioxane (4 M, 1.55 mmol in 0.39 mL) was added dropwise. The addition was performed under an argon atmosphere at 0 °C, and the reaction was stirred for 2 hours at 0 °C. The solvent was evaporated under vacuum, and the resulting salt 18 (30 mg, 98%) was obtained as a white solid, which was used in subsequent reactions without any further purification. 18: ESI-MS, positive mode: *m*/*z* calcd mass for C_12_H_19_FN_4_O_5_ [M + H]^+^ = 319.1412, was found 318.95.

### 4-Nitrophenyl (2-(2-(2-(2-(5-fluoro-2,6-dioxo-3,6-dihydropyrimidin-1(2*H*)-yl)acetamido) ethoxy)ethoxy)ethyl)carbamate, 19

To a solution of compound 18 (35 mg, 0.099 mmol) in dry dimethylformamide (1.5 mL) and DIPEA (38.3 mg, 0.296 mmol), 4-nitrophenyl chloroformate (26 mg, 0.128 mmol) was added and the reaction turns yellow in colour. The addition was performed under an argon atmosphere at 0 °C, and the reaction was stirred for 2 hours at room temperature. After complete consumption of the starting material, the solution containing the activated ester 19 was used directly in the next reaction without isolation. 19: ESI-MS, positive mode: *m*/*z* calcd mass for C_19_H_22_FN_5_O_9_ [M + H]^+^ = 484.1474, was found 484.00.

### (JH-VII-139-1)–(5-FU) conjugate, geo140

To a solution of JH-VII-139-1 (13 mg, 0.034 mmol), DIPEA (12 μL, 0.068 mmol) in dry dimethylformamide (0.75 mL), a crude solution of the activated ester 19 (1 equivalent) in dry dimethylformamide (0.75 mL) was added. The addition was performed under argon atmosphere, and the reaction was stirred for 24 hours at room temperature. After the complete consumption of JH-VII-139-1, the reaction was concentrated under vacuum, and the residue was subjected to column chromatography (ethyl acetate/methanol, gradient elution from 10 : 0.5 to 10 : 1) to obtain the final conjugate geo140 (4.9 mg, 20%) as a green, fluorescent solid. geo140: ^1^H NMR (500 MHz, DMSO-*d*_6_) *δ* 8.45–8.41 (m, 2H), 8.35 (d, *J* = 8.1 Hz, 1H), 8.29–8.24 (m, 1H), 8.16 (s, 1H), 8.04 (s, 1H), 8.00 (d, *J* = 6.8 Hz, 1H), 7.96 (s, 1H), 7.66–7.62 (m, 1H), 6.92 (d, *J* = 2.7 Hz, 1H), 5.42 (s, 1H), 4.26 (s, 2H), 3.61–3.45 (m, 8H), 3.43–3.38 (m, 2H), 3.24–3.19 (m, 2H), 2.93 (q, *J* = 7.5 Hz, 2H), 1.80 (s, 6H), 1.17 (t, *J* = 7.5 Hz, 3H). ^13^C NMR (126 MHz, DMSO-*d*_6_) *δ* 179.0, 166.6, 162.3, 160.4, 157.6, 153.0, 149.7, 149.5, 145.5, 140.8, 139.3, 135.8, 135.5, 131.2, 129.7, 128.1, 127.7, 126.3, 126.1, 125.1, 121.7, 120.7, 116.6, 116.5, 109.4, 104.9, 69.6, 69.5, 69.0, 68.7, 49.6, 48.6, 48.5, 36.4, 29.9, 26.0, 15.2. ESI-MS, positive mode: *m*/*z* calcd mass for C_37_H_37_FN_8_O_7_ [M + H]^+^ = 725.2842, was found 724.85.

### Biological experiments

Intestinal myofibroblast isolation: primary intestinal subepithelial myofibroblasts were isolated from freshly collected intestinal mucosa biopsies as previously described,^28^ from healthy individuals undergoing endoscopy for reasons of surveillance, after obtaining written and informed consent. Briefly, 4–6 pieces of intestinal tissue were collected in ice-cold Hank's Balanced Salt Solution (HBSS; Biosera, Nuaille, France) containing Ca^2+^/Mg^2+^ supplemented with P/S/A/G: penicillin (100 U ml^−1^), streptomycin (100 μg ml^−1^), amphotericin (2.5 μg ml^−1^) and gentamycin (50 μg ml^−1^), followed by 15 minute washes with HBSS with and without Ca^2+^/Mg^2+^ to remove possible contaminants. Next tissue was de-epithelialized by a 15 minute incubation in HBSS without Ca^2+^/Mg^2+^ containing 1 mM dithiothreitol (DTT, Sigma-Aldrich, Darmstadt, Germany) followed by 3× 30 minute washes in HBSS supplemented with 1 mM ethylenediaminetetraacetic acid (EDTA, Sigma-Aldrich, Darmstadt, Germany) at 37 °C. Tissue was cultured in RPMI 1640 (PAN Biotech, Aidenbach, Germany) supplemented with 10% fetal bovine serum (FBS; Biosera, Nuaille, France) and P/S/A/G for as long as 30 days at 37 °C and 5% CO_2_. After colonies of myofibroblasts reached 30% confluency, cells were detached and consequently cultured in 75 cm^2^ flasks in Dulbecco's modified Eagle medium (DMEM, 4.5 g l^−1^ glucose; PAN Biotech, Aidenbach, Germany) with 10% fetal bovine serum and P/S/A.

### Cell culture

HeLa, K562, HT-29, Caco-2, HepG2 and A549 cancer cell lines were obtained from the American Type Culture Collection (ATCC). All cell lines were cultured at 37 °C with 5% CO_2_. HeLa, Caco-2, HepG2 and A549 cells were maintained in Dulbecco's modified Eagle medium (DMEM, PAN Biotech) supplemented with 10% FBS and antibiotics/antimycotics, while K562 cells were maintained in RPMI and HT-29 cells in McCoy's 5a medium (Biosera) with the same supplements.

### Viability assays

All cells were seeded in 96-well plates (Corning, New York, USA) at density of 10^4^ cells per well, except HeLa that were seeded at destiny of 2 × 10^3^. After 24 hours, the cells were treated with increasing concentrations of the inhibitory compounds for 48 hours. Cytotoxic activity of parent and conjugate compounds was assessed by 3-(4,5-dimethylthiazol-2-yl)-2,5-diphenyltetrazolium bromide (MTT) assay, according to manufacturer's instructions. In brief, 100 μl of growth medium containing 0.5 mg ml^−1^ MTT (Sigma-Aldrich, Darmstadt, Germany) were added to each well of a 96-well plate and incubated at 37 °C and 5% CO_2_ for 2 hours until the formation of visible intracellular purple crystals. Then, MTT-supplemented growth medium was discarded and 100 μl dimethyl sulfoxide (Sigma-Aldrich, Darmstadt, Germany) were added per well for solubilization of dye. Absorbance was then measured on a microplate reader (Diareader EL 800; Dialab, Wr. Neudorf, Austria) at 570 nm with a reference wavelength at 660 nm.

### *In vitro* kinase assays

Human SRPK1, SRPK2, and a turkey LBR N-terminal fragment (amino acids 62–92; LBRNt(62–92)) were expressed as GST-fusion proteins in bacteria using the pGEX-2T vector (Amersham Biosciences) as previously described.^15^ The GST-fusion proteins were purified with glutathione-sepharose resin (Amersham Biosciences) following the manufacturer's protocol. Kinase assays (25 μL total volume) contained 0.5 μg GST-SRPK1 or GST-SRPK2, 1 μg GST-LBRNt(62–92) as substrate, 12 mM Hepes pH 7.5, 10 mM MgCl_2_, 25 μM ATP, and varying concentrations of inhibitors as indicated. Phosphorylated GST-LBRNt(62–92) was detected by autoradiography (Super RX film, Fujifilm). Incorporated radioactivity was quantified by scintillation counting of the excised SDS-PAGE gel bands.

### Immunofluorescence

HeLa cells (3 × 10^4^ cells per well) were seeded on glass coverslips in 24-well plates. After 24 hours, cells were treated with the indicated concentrations of inhibitors for 24 or 48 hours. Following incubation, samples were fixed with 4% paraformaldehyde in PBS for 20 minutes at room temperature. Paraformaldehyde was quenched with 100 mM Tris-HCl (pH 7.5), and cells were permeabilized with 0.2% Triton X-100 in PBS for 10 minutes. Blocking was performed with 0.5% fish skin gelatin in PBS. Cells were then probed with a primary antibody (anti-SRPK1 monoclonal, 1 : 100 dilution, BD Biosciences, San Jose, CA, USA) and a secondary antibody (Alexa488-conjugated goat anti-mouse, 1 : 400 dilution, Molecular Probes, Eugene, OR, USA), followed by DNA staining with propidium iodide, as previously described.^25^ Coverslips were mounted with mounting medium (0.01% *p*-phenylenediamine and 50% glycerol in PBS) and visualized using a Nikon confocal microscope with EZ-C1 3.20 software.

## Author contributions

V. S., E. N. and G. L. conceived the study. G. L. synthesized the compounds. G. L. performed protein mass spectrometry experiments, HPLC analysis and stability studies. I. S. carried out biochemical kinase assays, I. S., G. K., M. S., E. N., T. G. carried out and supervised the cytotoxicity screening, V. S., T. G., E. N. and G. K. acquired funding to carry out the project. G. L., V. S., E. N., T. G. wrote the manuscript, and all authors contributed to manuscript editing.

## Conflicts of interest

The other authors declare no competing interests.

## Supplementary Material

MD-016-D5MD00731C-s001

## Data Availability

Supplementary information is available. See DOI: https://doi.org/10.1039/D5MD00731C. The data supporting this article are available in its supplementary information (SI). The supplementary information (SI) contains copies of ^1^H and ^13^C NMR spectra, 2D NMR spectra, MS spectra, and analytical HPLC traces for all final compounds.

## References

[cit1] Zhou Z., Fu X. D. (2013). Regulation of splicing by SR proteins and SR protein-specific kinases. Chromosoma.

[cit2] Nikolakaki E., Sigala I., Giannakouros T. (2022). Good cop, bad cop: The different roles of SRPKs. Front. Genet..

[cit3] Hogg E. K. J., Findlay G. M. (2023). Functions of SRPK, CLK and DYRK kinases in stem cells, development, and human developmental disorders. FEBS Lett..

[cit4] Nikas I. P., Themistocleous S. C., Paschou S. A., Tsamis K. I., Ryu H. S. (2019). Serine-Arginine Protein Kinase 1 (SRPK1) as a prognostic factor and potential therapeutic target in cancer: Current evidence and future perspectives. Cells.

[cit5] Duggan W. P., O'Connell E., Prehn J. H. M., Burke J. P. (2022). Serine-Arginine Protein Kinase 1 (SRPK1): a systematic review of its multimodal role in oncogenesis. Mol. Cell. Biochem..

[cit6] Bullock N., Oltean S. (2017). The many faces of SRPK1. J. Pathol..

[cit7] Nowak D. G., Amin E. M., Rennel E. S., Hoareau-Aveilla C., Gammons M., Damodoran G., Hagiwara M., Harper S. J., Woolard J., Ladomery M. R., Bates D. O. (2010). Regulation of vascular endothelial growth factor (VEGF) splicing from pro-angiogenic to anti-angiogenic isoforms: a novel therapeutic strategy for angiogenesis. J. Biol. Chem..

[cit8] Amin E. M., Oltean S., Hua J., Gammons M. V., Hamdollah-Zadeh M., Welsh G. I., Cheung M. K., Ni L., Kase S., Rennel E. S., Symonds K. E., Nowak D. G., Royer-Pokora B., Saleem M. A., Hagiwara M., Schumacher V. A., Harper S. J., Hinton D. R., Bates D. O., Ladomery M. R. (2011). WT1 mutants reveal SRPK1 to be a downstream angiogenesis target by altering VEGF splicing. Cancer Cell.

[cit9] Wang P., Zhou Z., Hu A., Ponte de Albuquerque C., Zhou Y., Hong L., Sierecki E., Ajiro M., Kruhlak M., Harris C., Guan K. L., Zheng Z. M., Newton A. C., Sun P., Zhou H., Fu X. D. (2014). Both decreased and increased SRPK1 levels promote cancer by interfering with PHLPP-mediated dephosphorylation of Akt. Mol. Cell.

[cit10] Fukuhara T., Hosoya T., Shimizu S., Sumi K., Oshiro T., Yoshinaka Y., Suzuki M., Yamamoto N., Herzenberg L. A., Herzenberg L. A., Hagiwara M. (2006). Utilization of host SR protein kinases and RNA-splicing machinery during viral replication. Proc. Natl. Acad. Sci. U. S. A..

[cit11] Batson J., Toop H. D., Redondo C., Babaei-Jadidi R., Chaikuad A., Wearmouth S. F., Gibbons B., Allen C., Tallant C., Zhang J., Du C., Hancox J. C., Hawtrey T., Da Rocha J., Griffith R., Knapp S., Bates D. O., Morris J. C. (2017). Development of potent, selective SRPK1 inhibitors as potential topical therapeutics for neovascular eye disease. ACS Chem. Biol..

[cit12] Hatcher J. M., Wu G., Zeng C., Zhu J., Meng F., Patel S., Wang W., Ficarro S. B., Leggett A. L., Powell C. E., Marto J. A., Zhang K., Ki Ngo J. C., Fu X. D., Zhang T., Gray N. S. (2018). SRPKIN-1: A covalent SRPK1/2 inhibitor that potently converts VEGF from pro-angiogenic to anti-angiogenic isoform. Cell Chem. Biol..

[cit13] Thysiadis S., Katsamakas S., Dalezis P., Chatzisideri T., Trafalis D., Sarli V. (2017). Novel c(RGDyK)-based conjugates of POPAM and 5-fluorouracil for integrin-targeted cancer therapy. Future Med. Chem..

[cit14] Chatzisideri T., Leonidis G., Sarli V. (2018). Cancer-targeted delivery systems based on peptides. Future Med. Chem..

[cit15] Leonidis G., Dalezis P., Trafalis D., Beis D., Giardoglou P., Koukiali A., Sigala I., Nikolakaki E., Sarli V. (2021). Synthesis and Biological Evaluation of a c(RGDyK) Peptide Conjugate of SRPIN803. ACS Omega.

[cit16] Leonidis G., Koukiali A., Sigala I., Tsimaratou K., Beis D., Giannakouros T., Nikolakaki E., Sarli V. (2023). Synthesis and anti-angiogenic activity of novel c(RGDyK) peptide-based JH-VII-139-1 conjugates. Pharmaceutics.

[cit17] Greenwald R. B., Pendri A., Conover C. D., Zhao H., Choe Y. H., Martinez A., Shum K., Guan S. (1999). Drug delivery systems employing 1,4- or 1,6-elimination: poly(ethylene glycol) prodrugs of amine-containing compounds. J. Med. Chem..

[cit18] Jeffrey S. C., Torgov M. Y., Andreyka J. B., Boddington L., Cerveny C. G., Denny W. A., Gordon K. A., Gustin D., Haugen J., Kline T., Nguyen M. T., Senter P. D. (2005). Design, synthesis, and in vitro evaluation of dipeptide-based antibody minor groove binder conjugates. J. Med. Chem..

[cit19] Chatzisideri T., Leonidis G., Karampelas T., Skavatsou E., Velentza-Almpani A., Bianchini F., Tamvakopoulos C., Sarli V. (2022). Integrin-Mediated Targeted Cancer Therapy Using c(RGDyK)-Based Conjugates of Gemcitabine. J. Med. Chem..

[cit20] Voukkalis N., Koutroumani M., Zarkadas C., Nikolakaki E., Vlassi M., Giannakouros T. (2016). SRPK1 and Akt Protein Kinases phosphorylate the RS domain of Lamin B Receptor with distinct specificity: A combined biochemical and In silico approach. PLoS One.

[cit21] Zhong X. Y., Ding J. H., Adams J. A., Ghosh G., Fu X. D. (2009). Regulation of SR protein phosphorylation and alternative splicing by modulating kinetic interactions of SRPK1 with molecular chaperones. Genes Dev..

[cit22] Edmond V., Moysan E., Khochbin S., Matthias P., Brambilla C., Brambilla E., Gazzeri S., Eymin B. (2011). Acetylation and phosphorylation of SRSF2 control cell fate decision in response to cisplatin. EMBO J..

[cit23] Vivarelli S., Lenzken S. C., Ruepp M. D., Ranzini F., Maffioletti A., Alvarez R., Mühlemann O., Barabino S. M. (2013). Paraquat modulates alternative pre-mRNA splicing by modifying the intracellular distribution of SRPK2. PLoS One.

[cit24] Sigala I., Koutroumani M., Koukiali A., Giannakouros T., Nikolakaki E. (2021). Nuclear translocation of SRPKs Is associated with 5-FU and cisplatin sensitivity in HeLa and T24 cells. Cells.

[cit25] Voskuhl J., Waller M., Bandaru S., Tkachenko B. A., Fregonese C., Wibbeling B., Schreiner P. R., Ravoo B. J. (2012). Nanodiamonds in sugar rings: An experimental and theoretical investigation of cyclodextrin-nanodiamond inclusion complexes. Org. Biomol. Chem..

[cit26] Vong K. K., Maeda S., Tanaka K. (2016). Propargyl-Assisted Selective Amidation Applied in C-terminal Glycine Peptide Conjugation. Chemistry.

[cit27] Yue K., Hou X., Jia G., Zhang L., Zhang J., Tan L., Wang X., Zhang Z., Li P., Xu W., Li X., Jiang Y. (2021). Design, synthesis and biological evaluation of hybrid of ubenimex-fluorouracil for hepatocellular carcinoma therapy. Bioorg. Chem..

[cit28] Filidou E., Valatas V., Drygiannakis I., Arvanitidis K., Vradelis S., Kouklakis G., Kolios G., Bamias G. (2018). Cytokine Receptor Profiling in Human Colonic Subepithelial Myofibroblasts: A Differential Effect of Th Polarization–Associated Cytokines in Intestinal Fibrosis. Inflammatory Bowel Dis..

