# Supplementary material for: Synthesis of novel SRPK1-targeting hybrid compounds incorporating antimetabolites for cancer therapy
Source: RSC Med Chem. 2025 Sep 29;16(12):6285–98. doi: 10.1039/d5md00731c (PMC12574689; doi:10.1039/d5md00731c)
Supplement: MD-016-D5MD00731C-s001 [file MD-016-D5MD00731C-s001.pdf]

Supplementary Information (SI) for RSC Medicinal Chemistry.
This journal is © The Royal Society of Chemistry 2025

SUPPLEMENTARY MATERIAL

# Synthesis of Novel SRPK1-Targeting Hybrid Compounds Incorporating Antimetabolites for Cancer Therapy

George Leonidis,[a,*] Ioanna Sigala,[a,*] Michail Spathakis,[b] George Kolios,[b] Thomas Giannakouros,[a] Eleni Nikolakaki[a] and Vasiliki Sarli[a,*]

[a] Department of Chemistry, Aristotle University of Thessaloniki, sarli@chem.auth.gr. [*] Author to whom correspondence should be addressed.

[b] Department of Medicine, Democritus University of Thrace.

## Supporting Information


| | |
|---|---|
| LC/ESI-MS, HPLC parameters and method development | S2 |
| $^{1}$H-NMR spectra for **6** | S3 |
| $^{1}$H-NMR spectra for **7** | S4 |
| $^{1}$H-NMR and $^{13}$C-NMR spectra for **9** | S5 |
| HSQC and HMBC spectra for **9** | S6 |
| LC/ESI-MS analysis of **9** | S7 |
| $^{1}$H-NMR and $^{13}$C-NMR spectra for **11** | S8 |
| ESI-MS analysis of **11** | S9 |
| $^{1}$H-NMR and $^{13}$C-NMR spectra for **geo15** | S10 |
| LC/ESI-MS analysis of **geo15** | S11 |
| $^{1}$H-NMR and $^{13}$C-NMR spectra for **14** | S12 |
| LC/ESI-MS analysis of **14** | S13 |
| $^{1}$H-NMR spectra for **17** | S14 |
| LC/ESI-MS analysis of **17** | S15 |
| $^{1}$H-NMR and $^{13}$C-NMR spectra for **geo140** | S16 |
| LC/ESI-MS analysis of **geo140** | S17 |
| Chemical stability data for **geo15** | S18 |
| Chemical stability data for **geo140** | S19 |

## HPLC-MS parameters and method development

Chromatographic separation was performed using a Shimadzu LC-20AD system coupled to a Shimadzu LCMS-2010EV mass spectrometer. A Supelco Discovery C18 column (250 × 4.6 mm, 5 μm particle size) was employed for the separation. The mobile phase was delivered at a flow rate of 0.4 mL/min, and the column temperature was maintained at 26 °C. UV detection was carried out at 254 nm. The mass spectrometer was operated at an ionization voltage of 1.65 kV. All compounds were eluted using gradient mixtures of water and acetonitrile (containing 0.01% formic acid) as the mobile phases, as described in the methods below.

Method 1:

**Table S1**. Conditions for Method 1.

| time (min) | $H_2O$ (% v/v conc.) | ACN (% v/v conc.) |
|---|---|---|
| 3 | 90 | 10 |
| 22 | 15 | 85 |
| 25 | 10 | 90 |
| 29 | 10 | 90 |
| 31 | 5 | 95 |
| 37 | 5 | 95 |

Method 2 (for **9**):

**Table S2**. Conditions for Method 2.

| time (min) | $H_2O$ (% v/v conc.) | ACN (% v/v conc.) |
|---|---|---|
| 3 | 90 | 10 |
| 25 | 20 | 80 |
| 30 | 10 | 90 |
| 38 | 10 | 90 |

Method 3 (for **geo15**):

**Table S3**. Conditions for Method 3.

| time (min) | $H_2O$ (% v/v conc.) | ACN (% v/v conc.) |
|---|---|---|
| 3 | 90 | 10 |
| 11 | 50 | 50 |
| 38 | 10 | 90 |

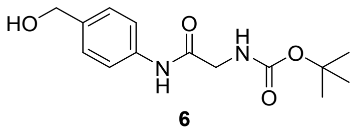


**6**

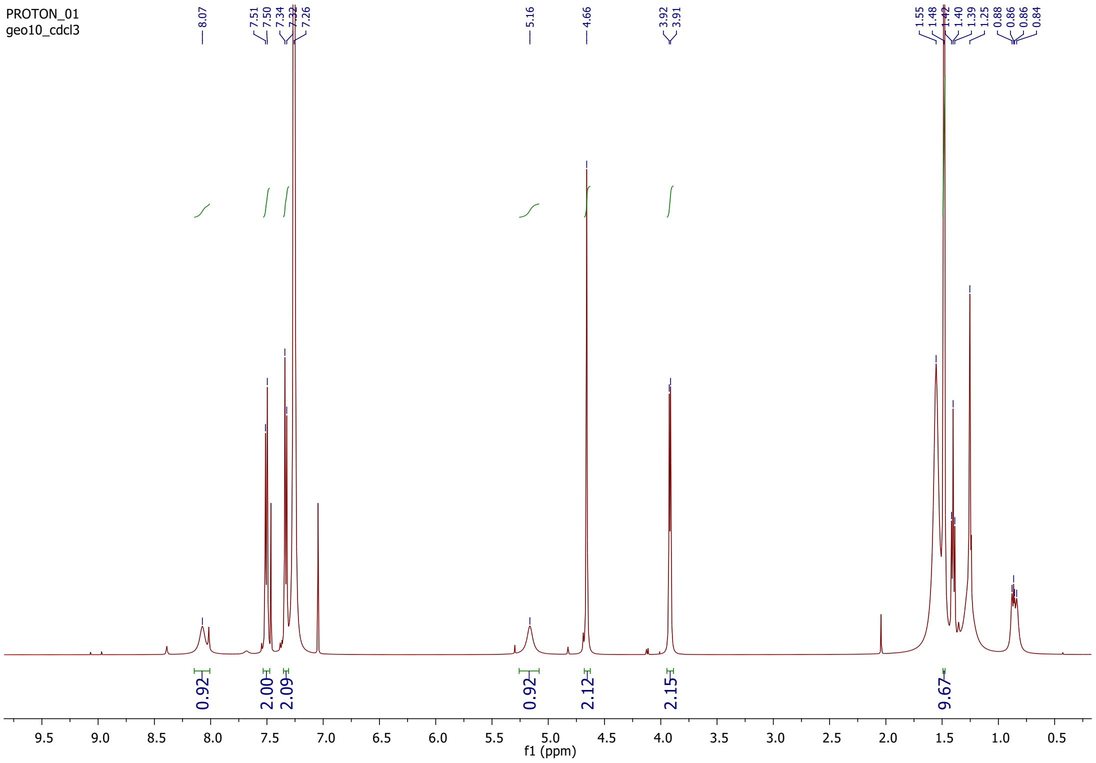


**Figure S1**. $^{1}$H-NMR spectra for **6**

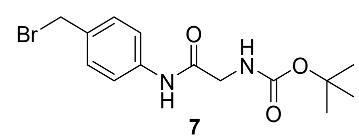

**7**

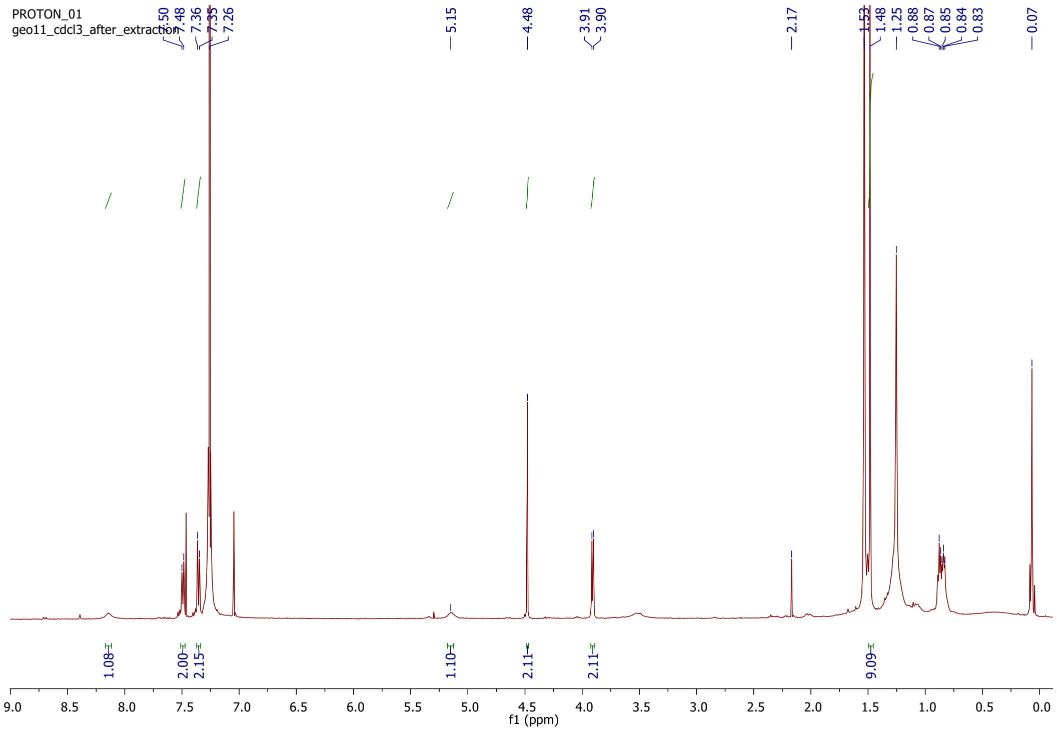


**Figure S2**. $^{1}$H-NMR spectra for **7**

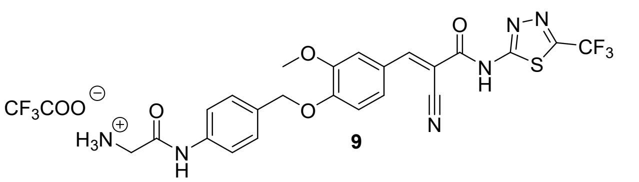


**9**

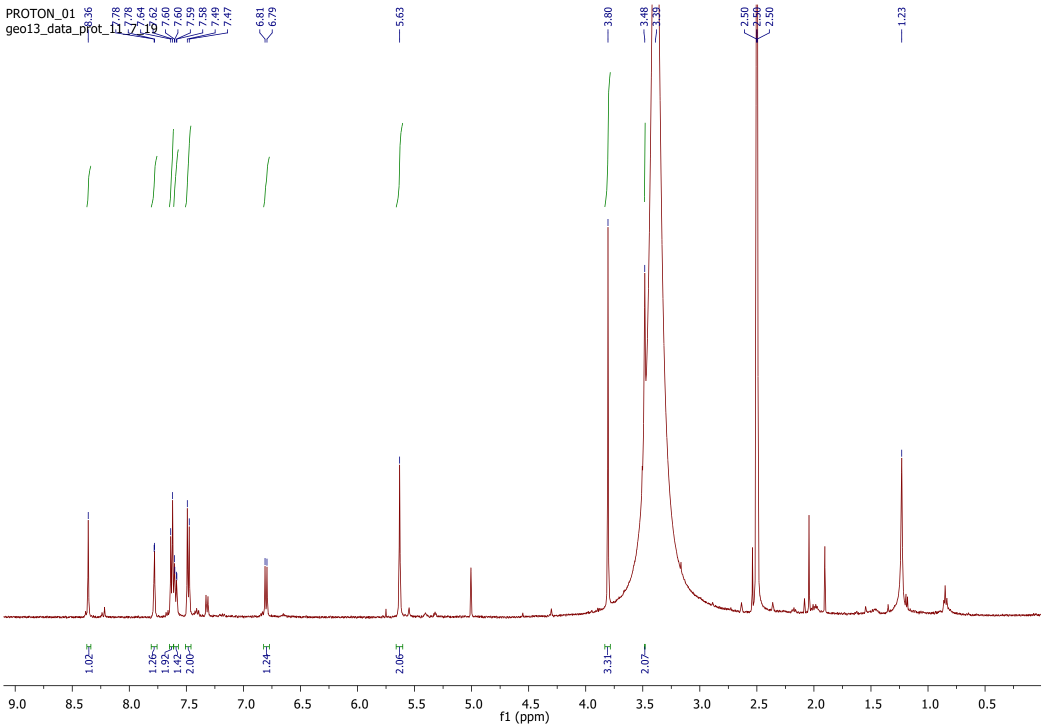


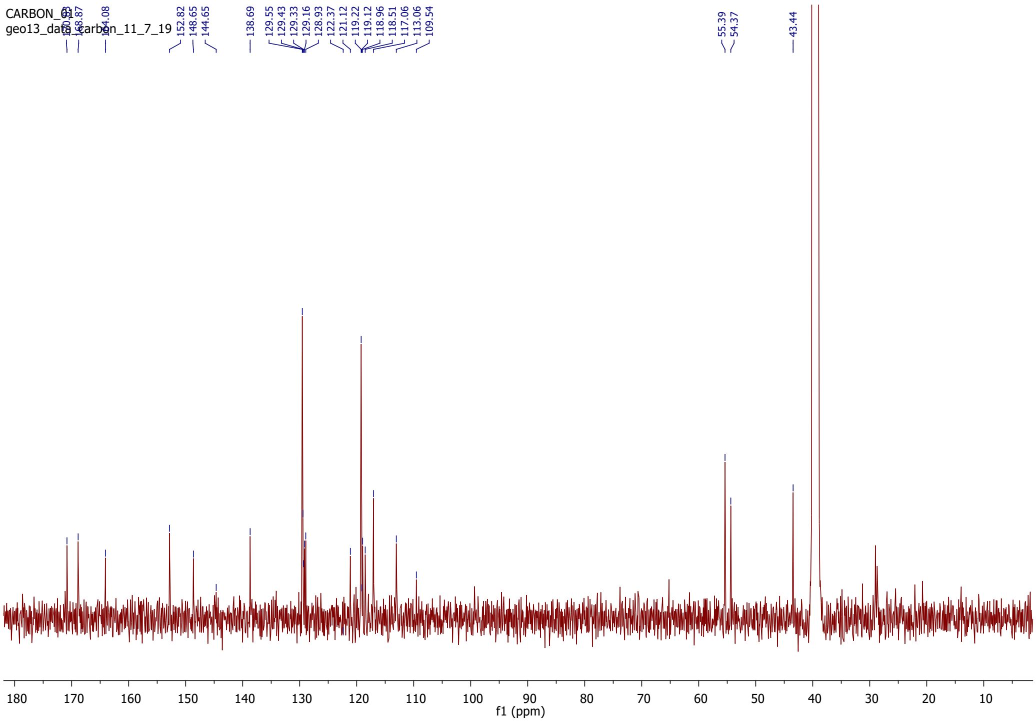


**Figure S3**. $^{1}H$-NMR and $^{13}C$-NMR spectra for **9**

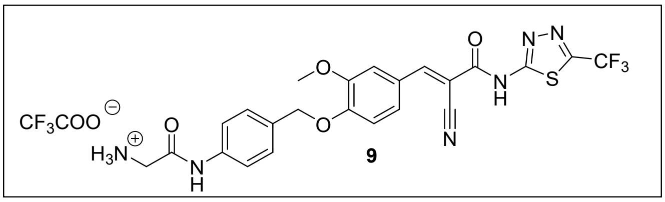


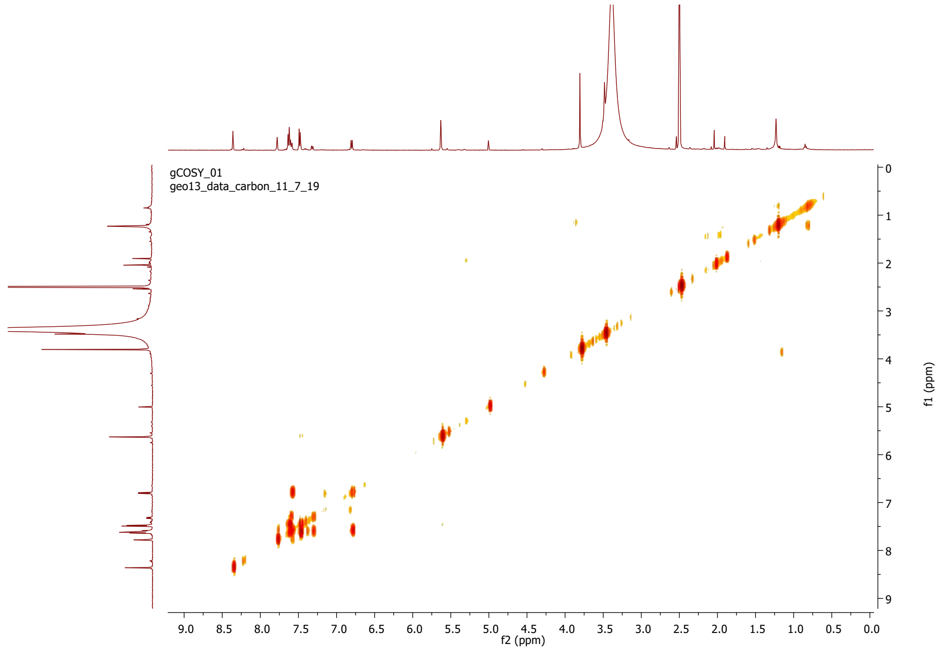


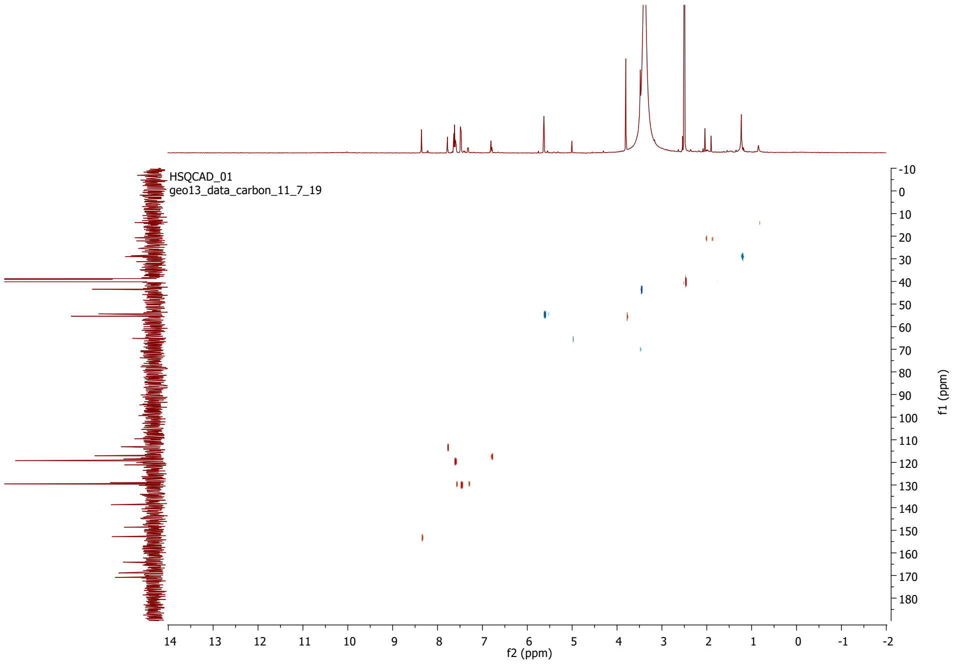


**Figure S4**. HSQC and HMBC spectra for **9**

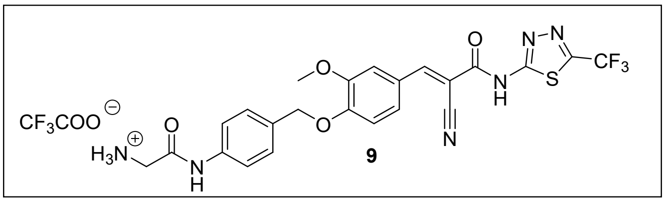


ESI-MS, positive mode: m/z calcd mass for $C_{25}H_{20}F_6N_6O_6S$ $[M-H]^-$ = 533.1213, was found 532.95.

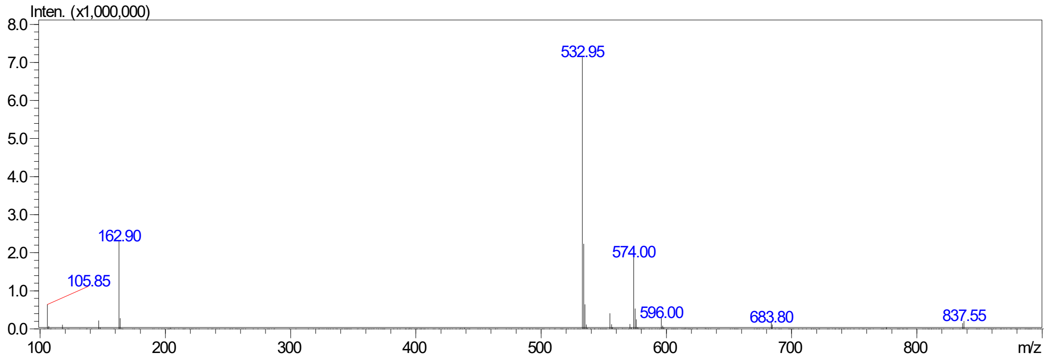


**Figure S5**: ESI-LCMS analysis for **9**.

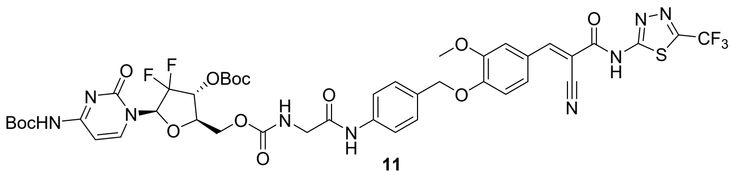

**11**

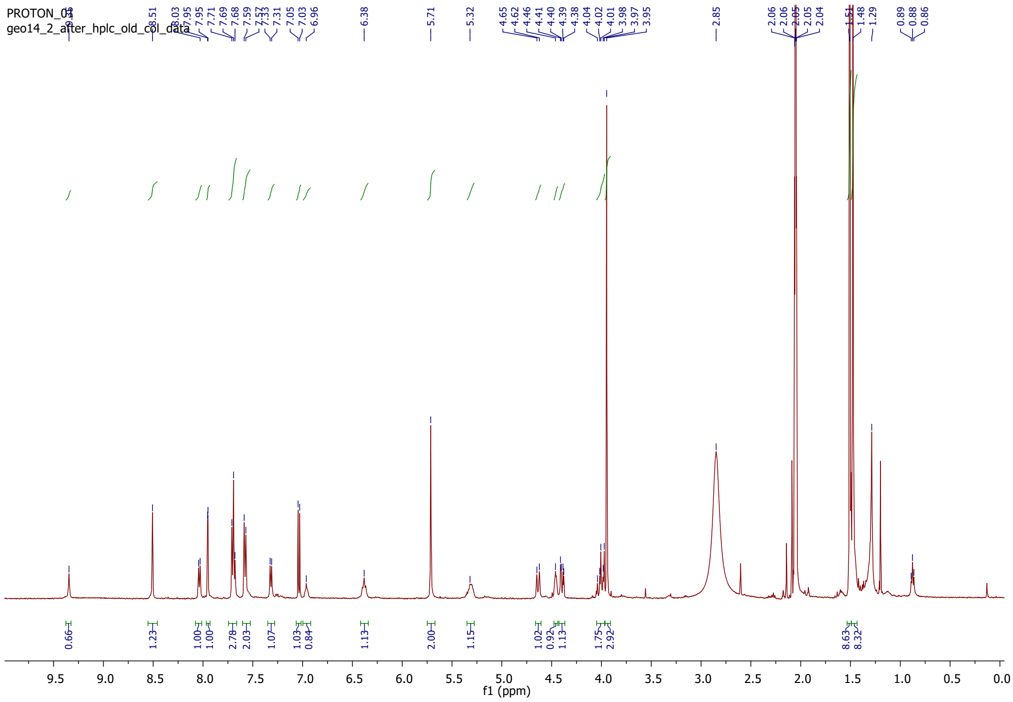

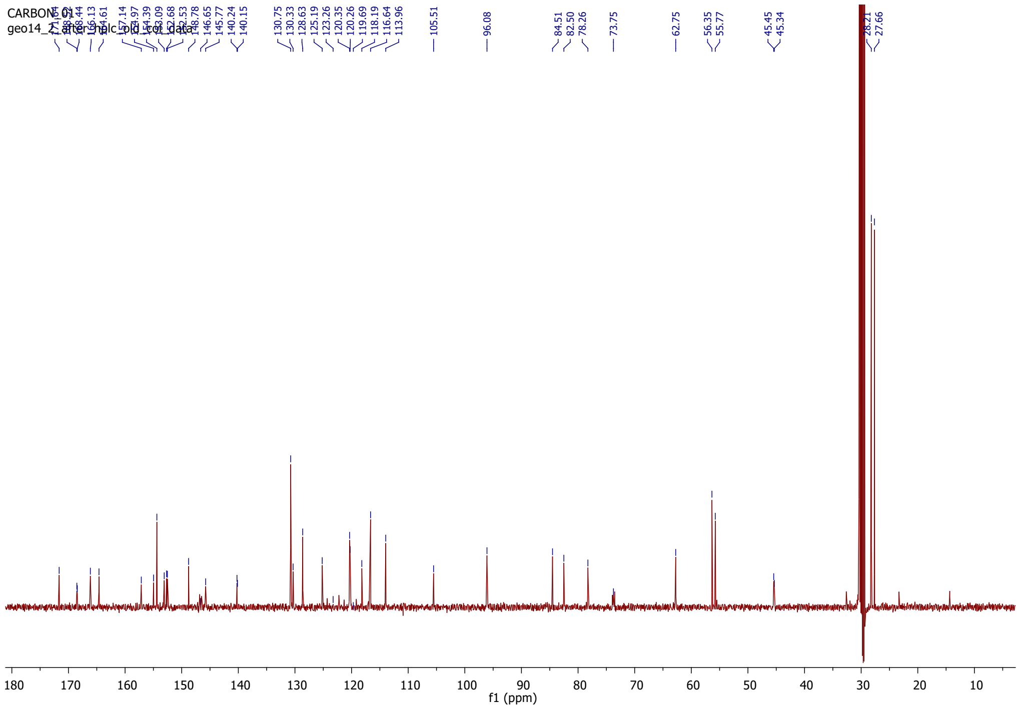

**Figure S6**. $^{1}$H-NMR and $^{13}$C-NMR spectra for **11**

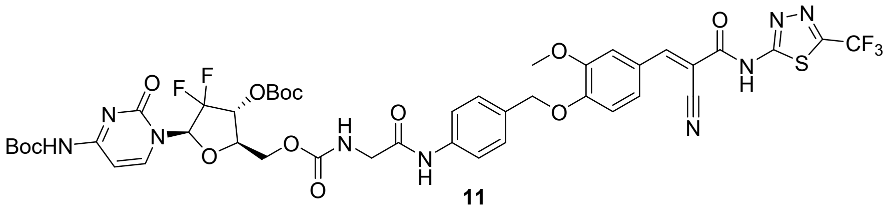


**11**

ESI-MS, negative mode: m/z calcd mass for $C_{43}H_{44}F_5N_9O_{13}S$ $[M-H]^-$ = 1020.2627, was found 1020.15

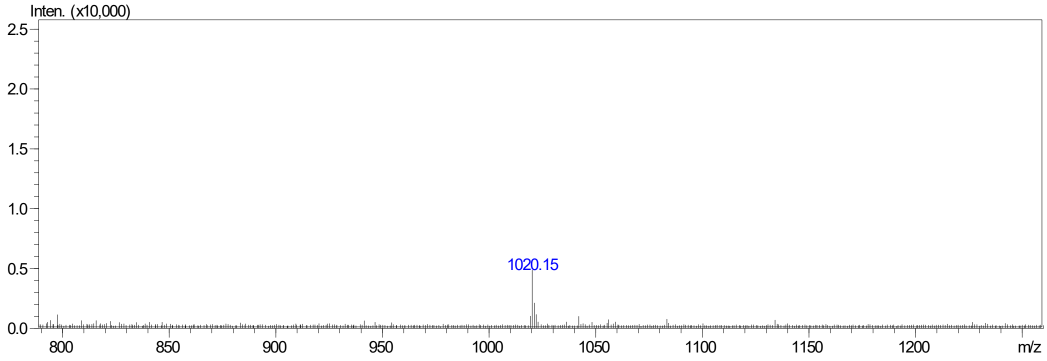


**Figure S7**: Mass Spectrometry analysis for **11**.

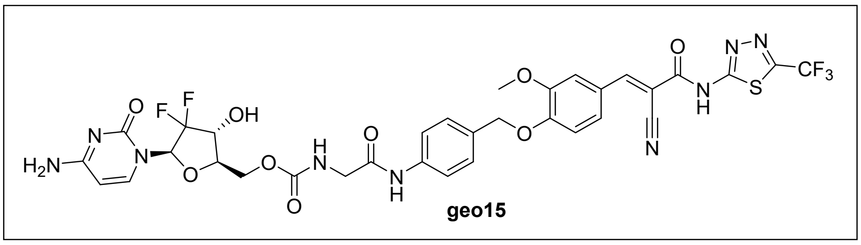


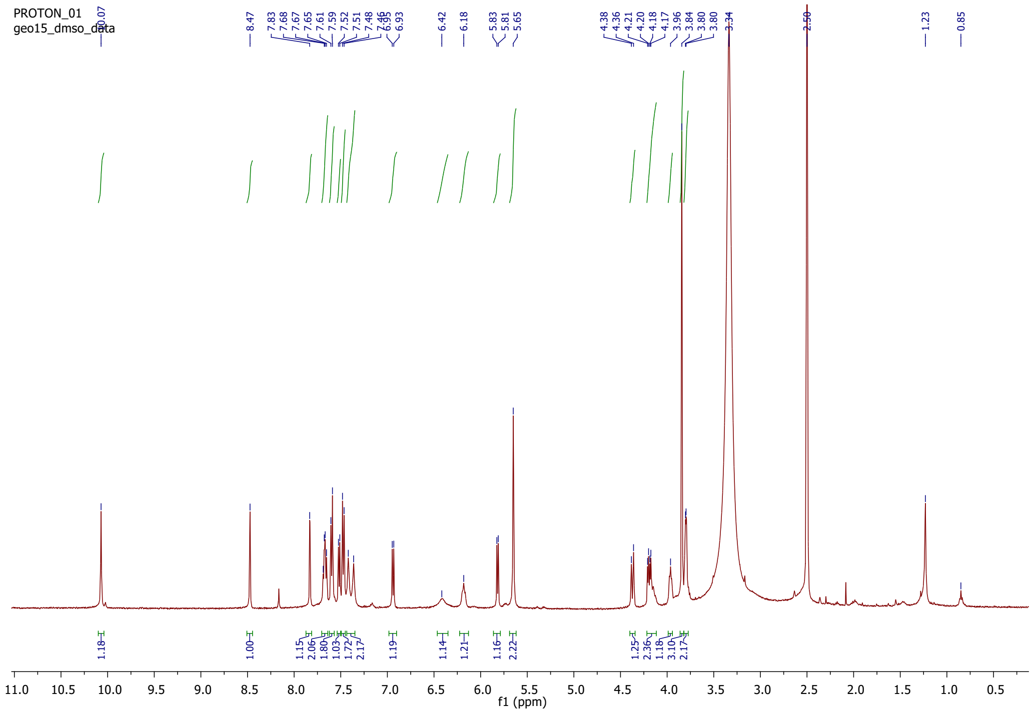

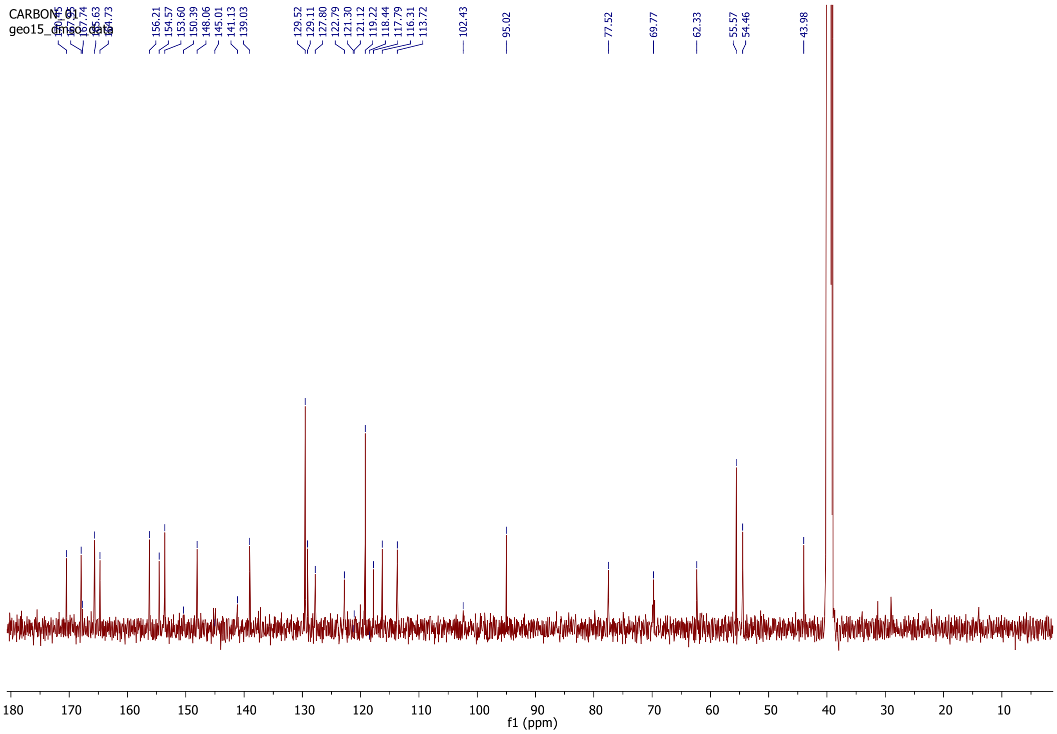

**Figure S8**. $^{1}$H-NMR and $^{13}$C-NMR spectra for **geo15**

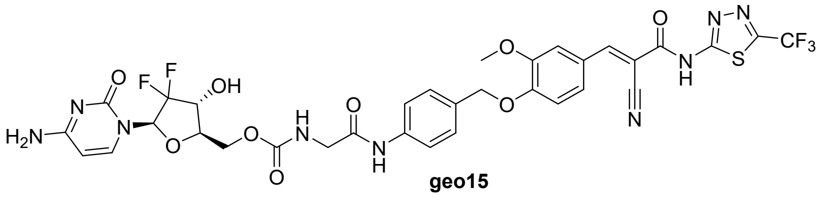


**geo15**

The compound **geo15** was eluted with method 3, with a retention time of 21.2 minutes. LC-MS analysis revealed a main peak with a relative area of 99.3 out of a total relative area of 100.0, corresponding to a calculated purity of 99.3%.

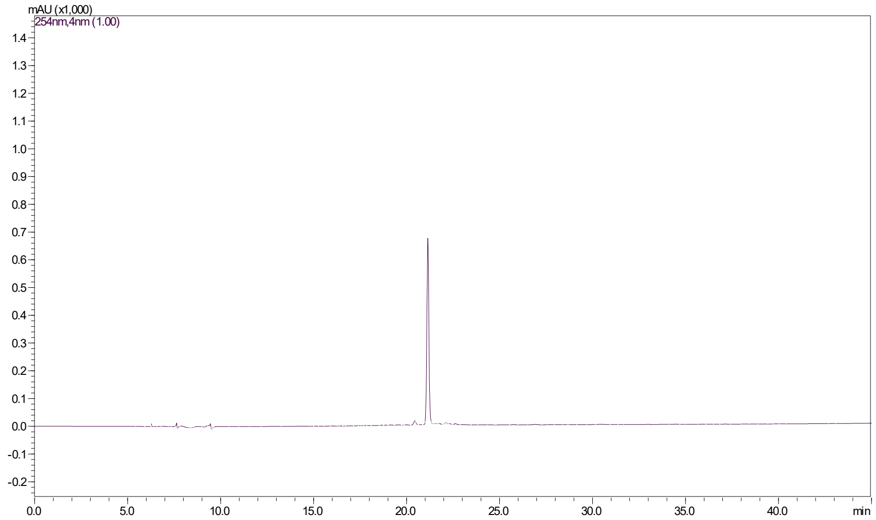


ESI-MS, positive mode: m/z calcd mass for $C_{33}H_{28}F_5N_9O_9S$ $[M+H]^+$ = 822.1724, was found 821.85

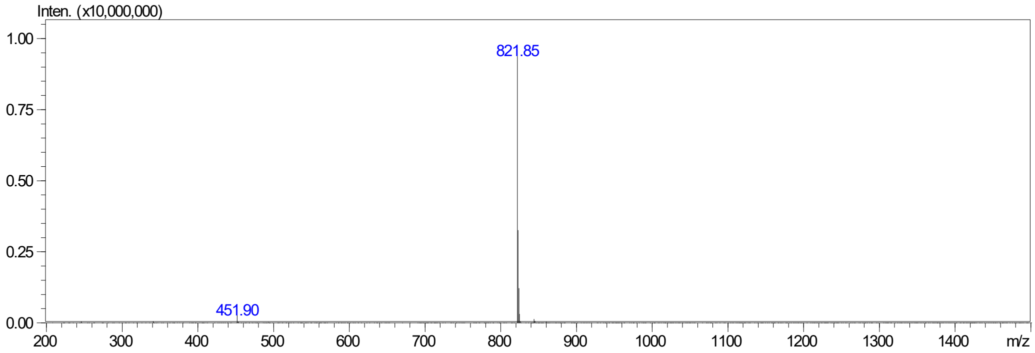


**Figure S9**: ESI-LCMS analysis for **geo15**.

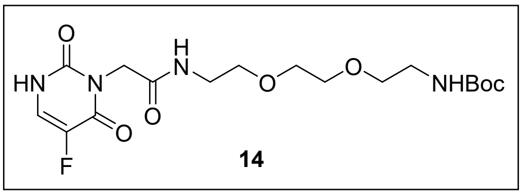

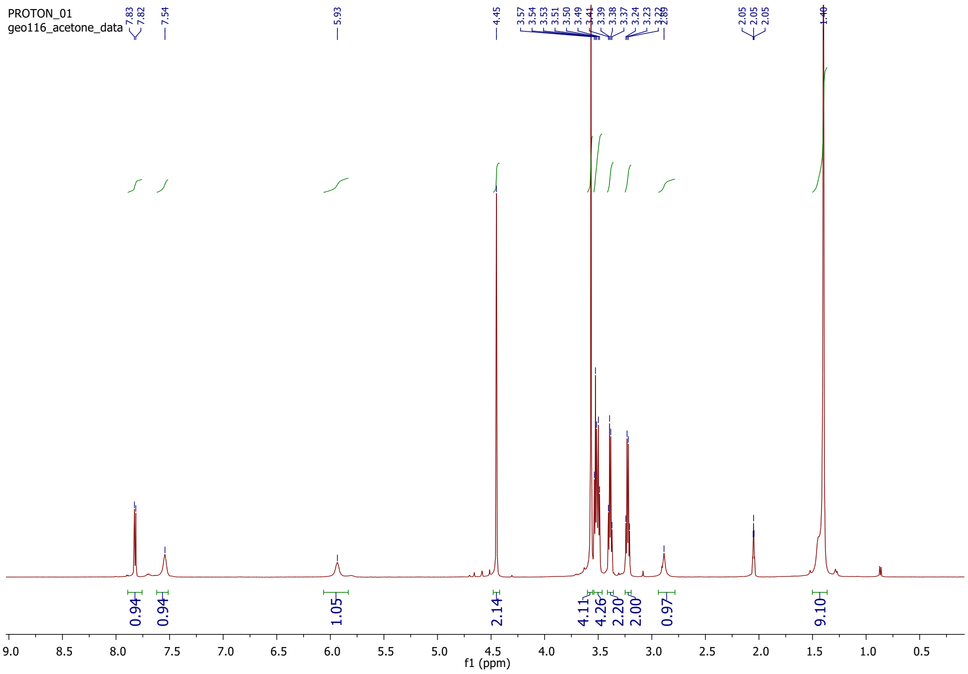

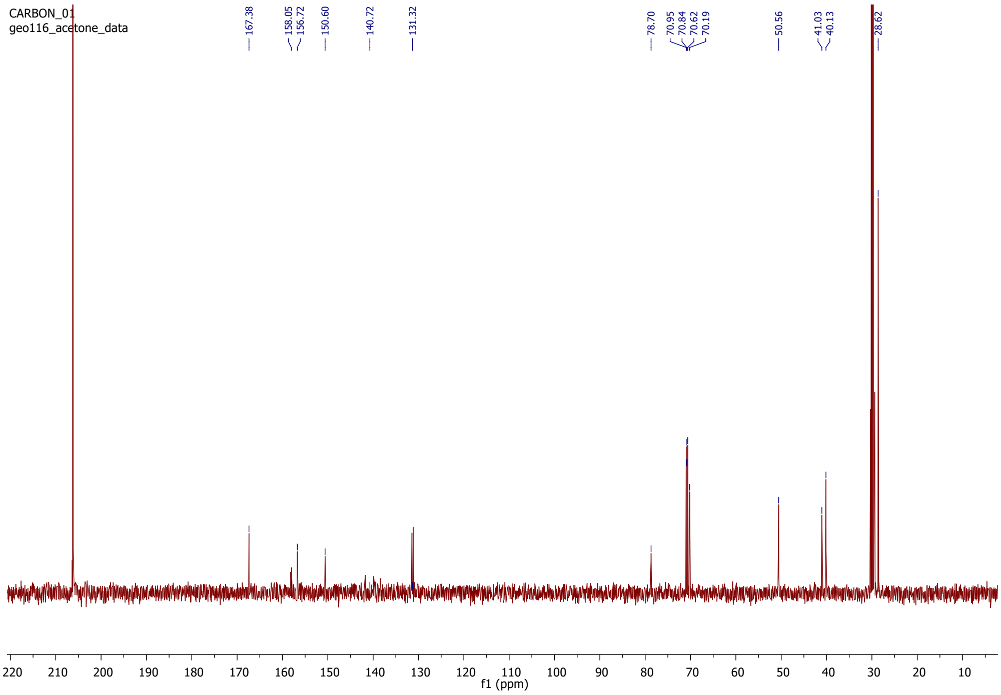

**Figure S10.** $^{1}$H-NMR and $^{13}$C-NMR spectra for **14**

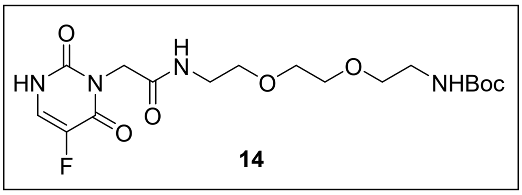


The compound **14** was eluted with method 1, with a retention time of 19.3 minutes. LC-MS analysis revealed a main peak with a relative area of 91.6 out of a total relative area of 100.0, corresponding to a calculated purity of 91.6%.

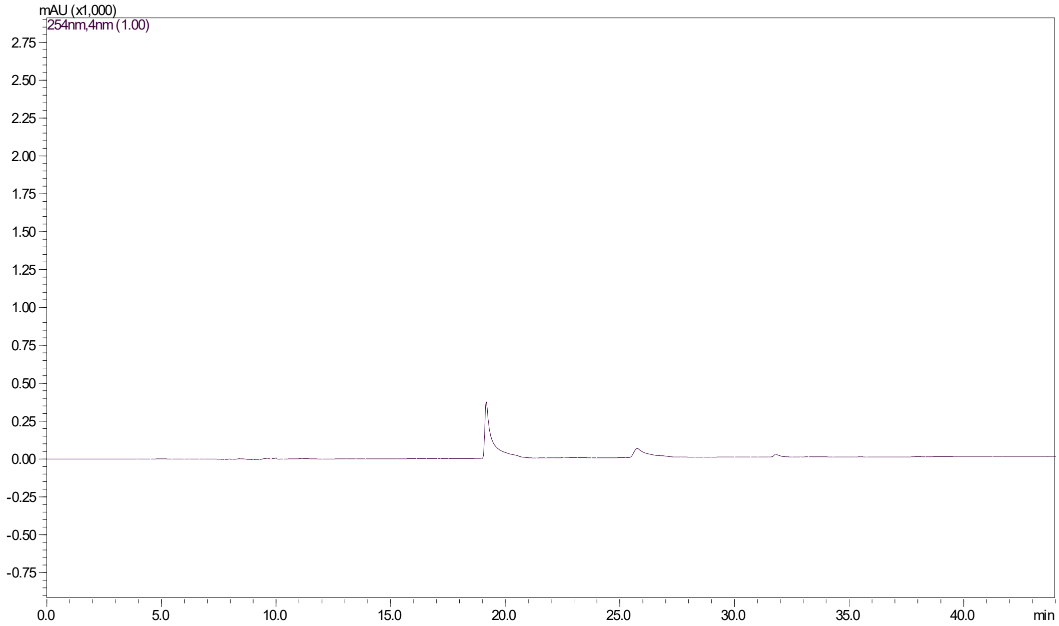


ESI-MS, negative mode: m/z calcd mass for $C_{17}H_{27}FN_4O_7$ $[M-H]^-$ = 417.1791, was found 416.95.

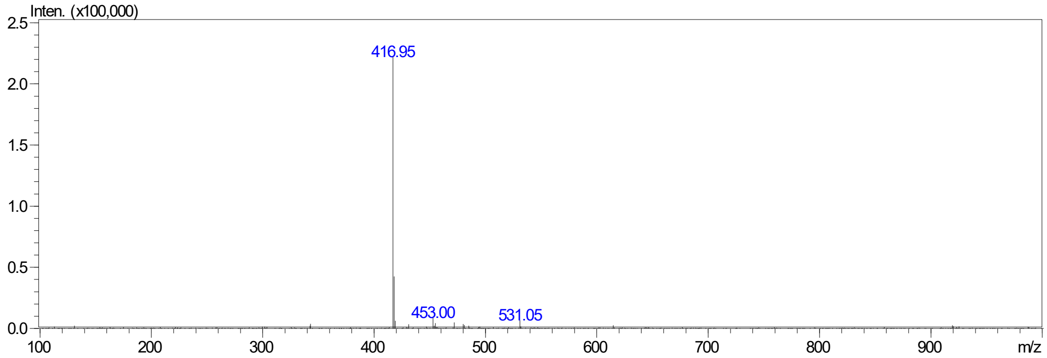


**Figure S11**: ESI-LCMS analysis for **14**

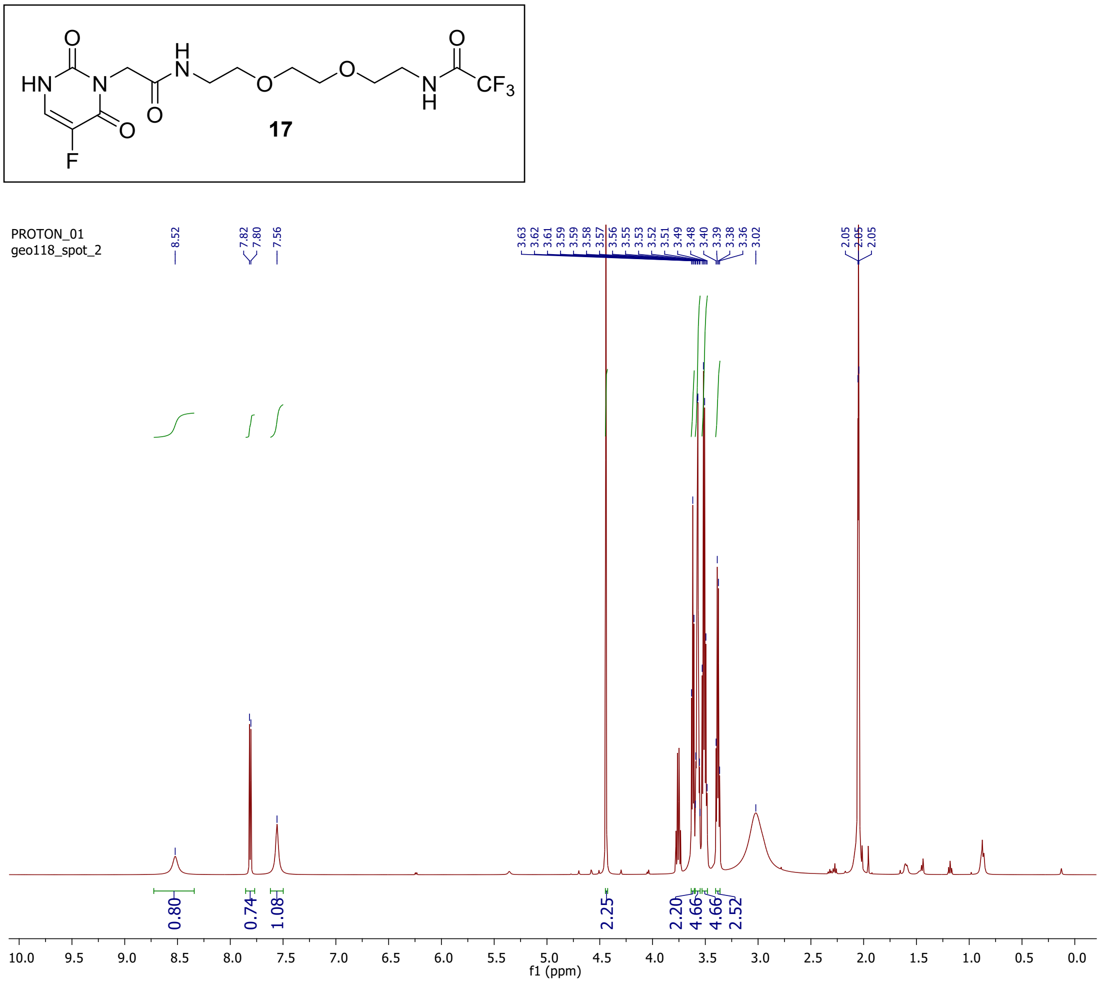


**Figure S12**. $^{1}$H-NMR spectra for **17**

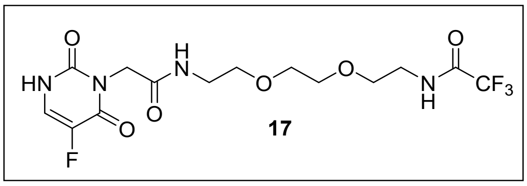


The compound **17** as eluted with method 1, with a retention time of 16.8 minutes. LC-MS analysis revealed a main peak with a relative area of 96.4 out of a total relative area of 100.0, corresponding to a calculated purity of 96.4%.

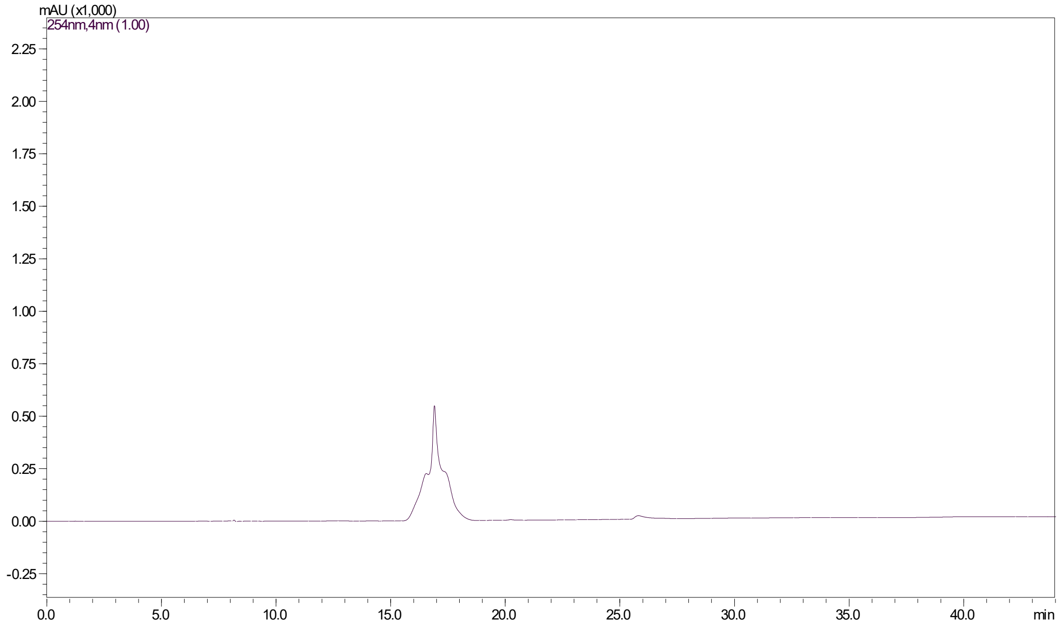


ESI-MS, negative mode: m/z calcd mass for $C_{14}H_{18}F_4N_4O_6$ $[M-H]^-$ = 413.1090, was found 416.95.

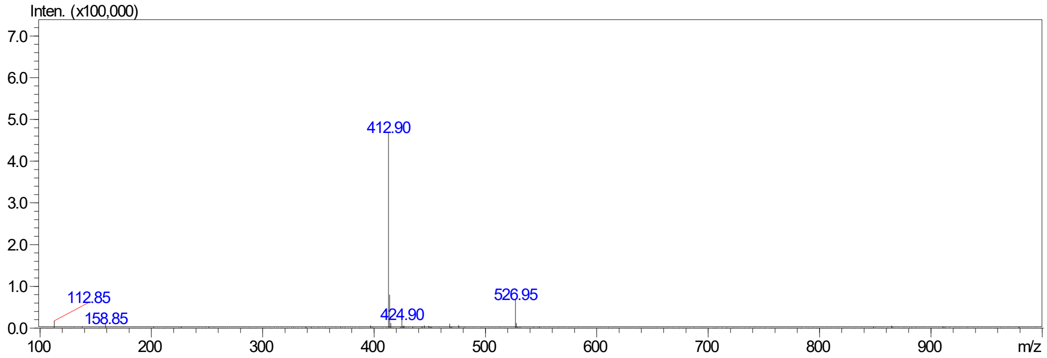


**Figure S13**: ESI-LCMS analysis for **17**.

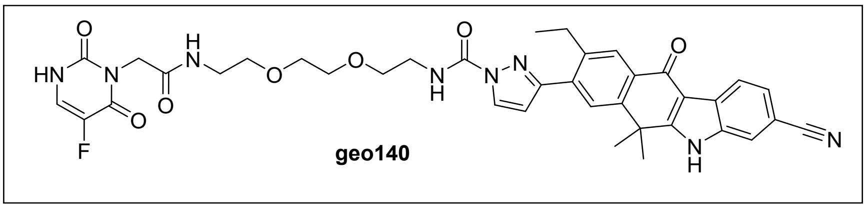


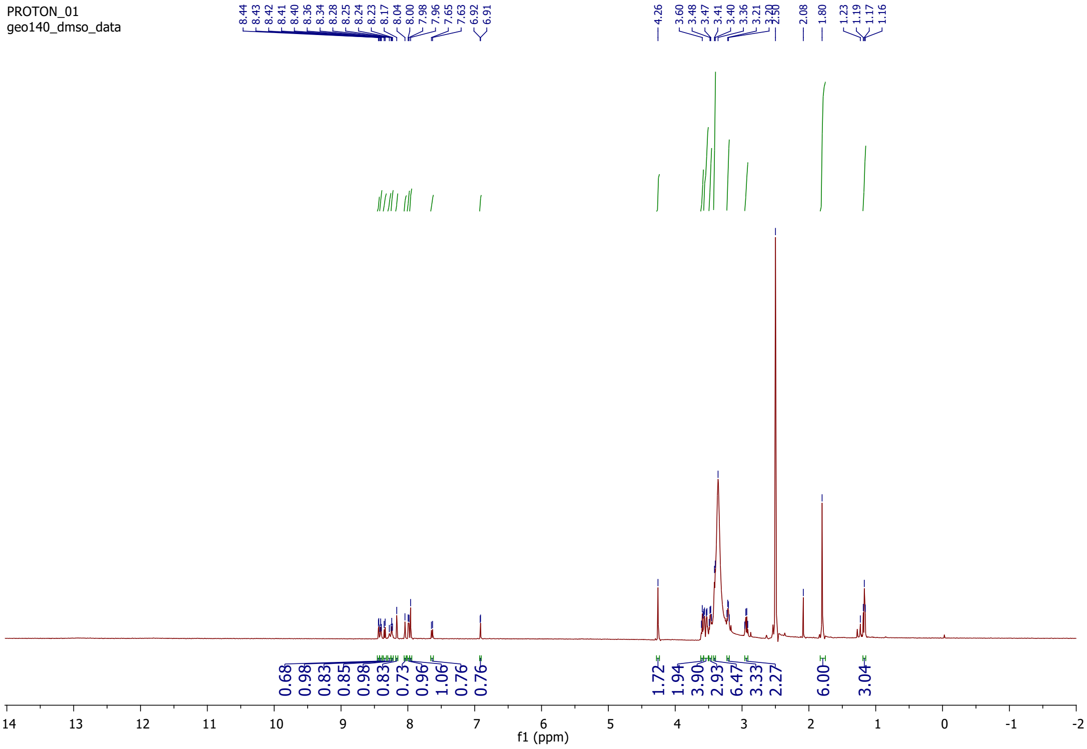


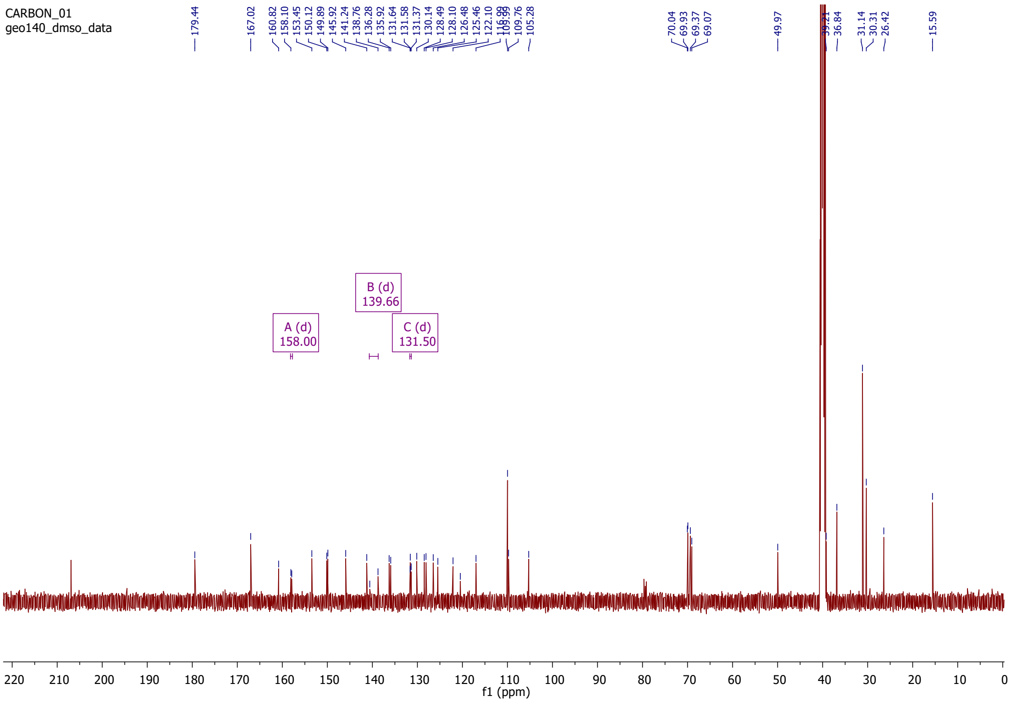


**Figure S14**. $^{1}$H-NMR and $^{13}$C-NMR spectra for **geo140**

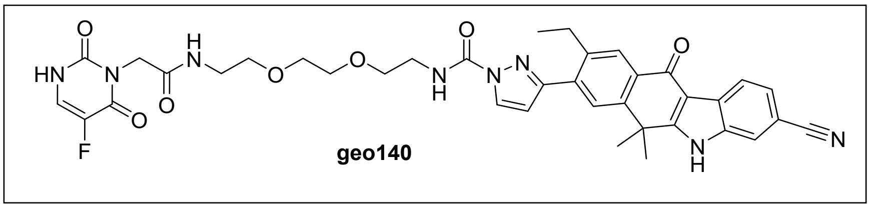


The compound **geo140** was eluted with method 1, with a retention time of 25.6 minutes. LC-MS analysis revealed a main peak with a relative area of 95.7 out of a total relative area of 100.0, corresponding to a calculated purity of 95.7%.

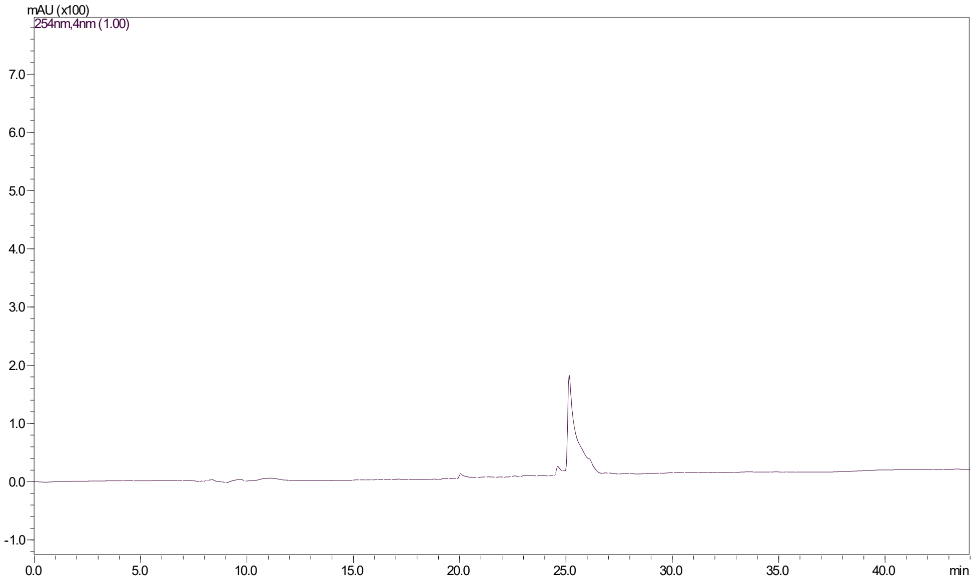


ESI-MS, negative mode: m/z calcd mass for $C_{37}H_{37}FN_8O_7$ $[M-H]^-$ = 723.2696, was found 722.95.

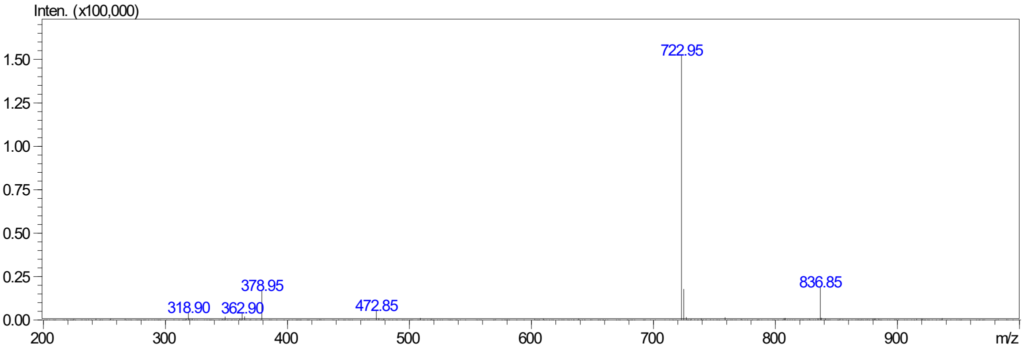


**Figure S15**: ESI-LCMS analysis for **geo140**.

## Chemical Stability Data

Chemical stability experiments were conducted using LC-MS Method 4, except for the pH stability studies of compound **geo15**, which were performed using LC-MS Method 3. Representative chromatograms are provided for each experiment. **geo15**:

pH = 5.2 t=0 and 24h

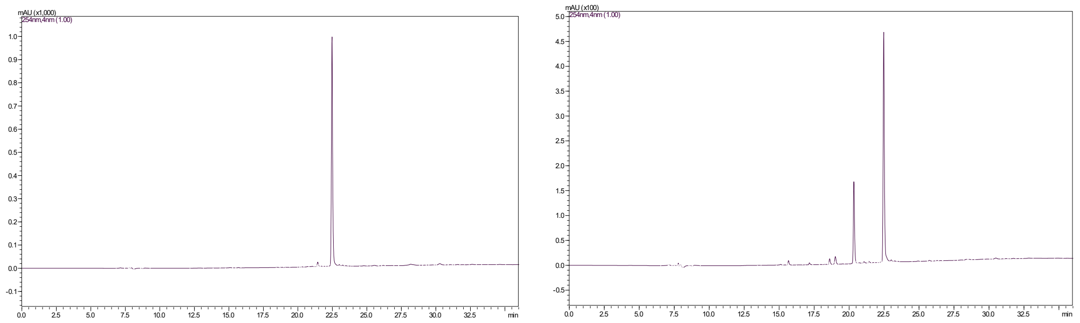

pH = 7.4 t=0 and 24h

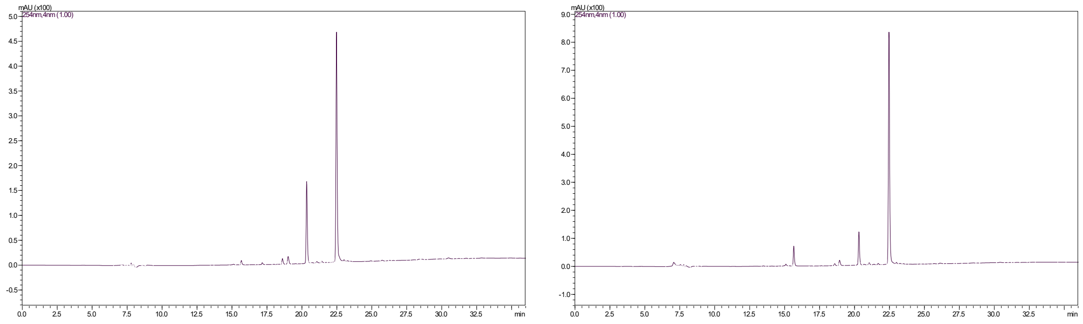

DMEM t=0 and 48h

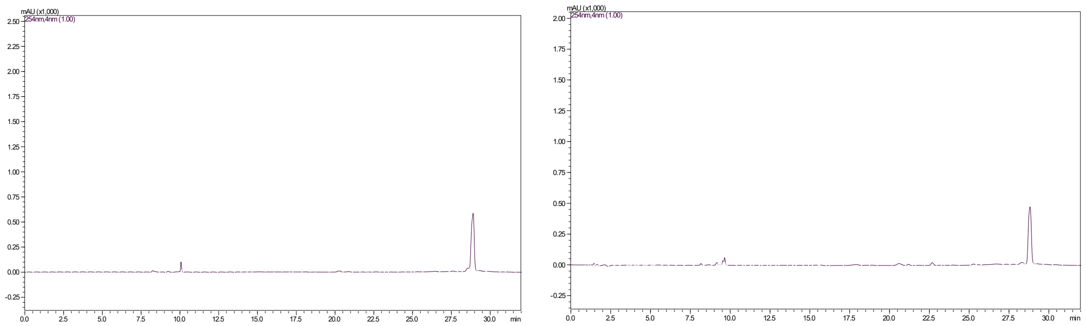

Plasma t=0 and 24h

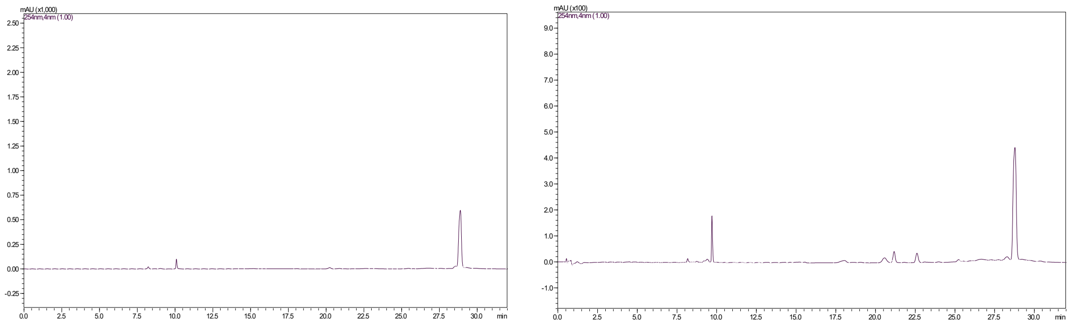

**Figure S16**. Chemical stability assay LC chromatograms for **geo15**.

**geo140**

pH = 5.2 t=0 and 24h

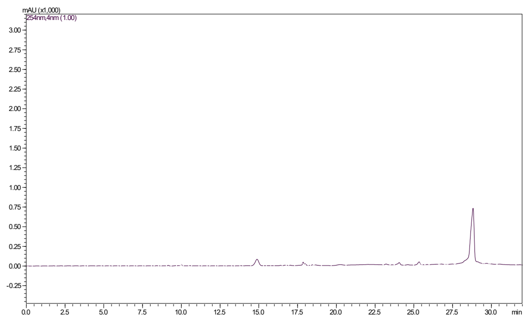

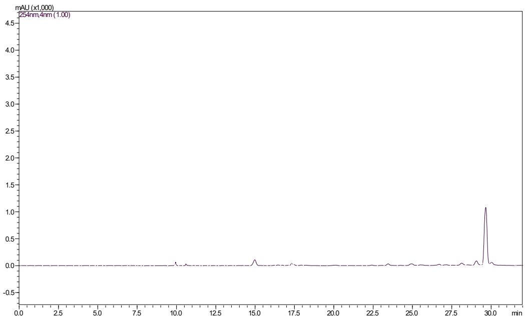

pH = 7.4 t=0 and 24h

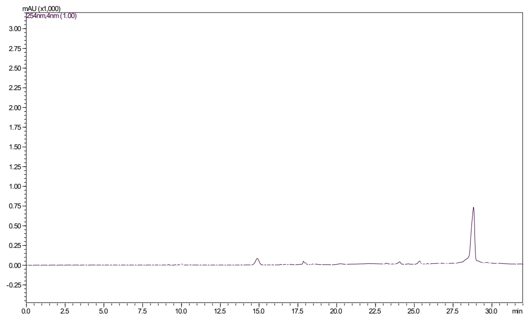

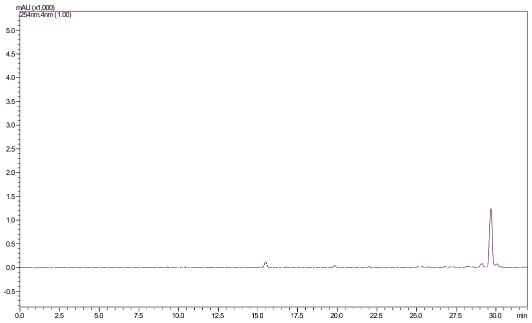

DMEM t=0 and 24h

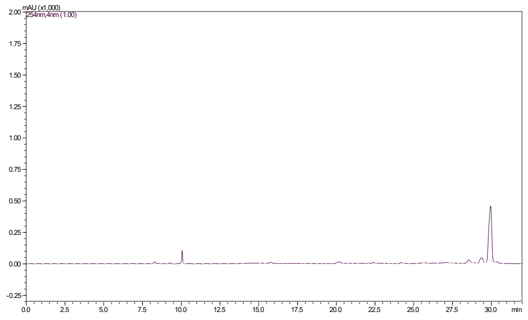

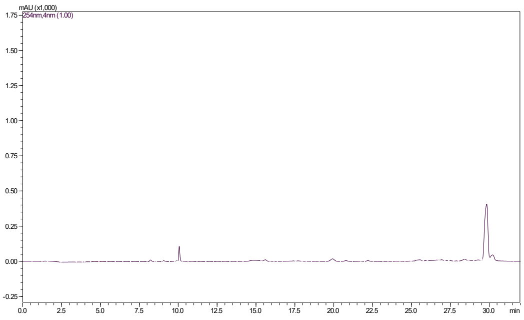

Plasma t=0 and 24h

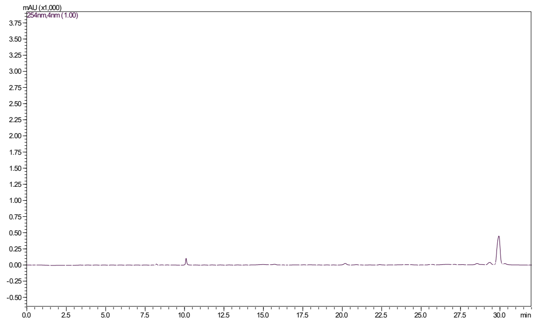

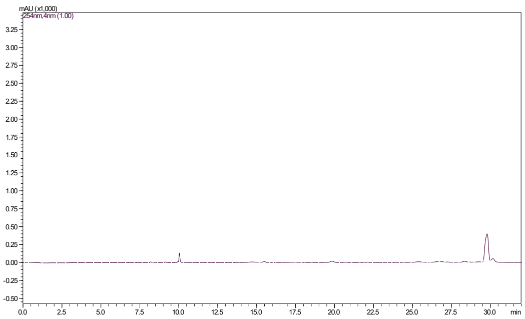

**Figure S17**. Chemical stability assay LC chromatograms for **geo140**.
